# Analysis of Endophytic Bacterial Diversity From Different *Dendrobium* Stems and Discovery of an Endophyte Produced Dendrobine-Type Sesquiterpenoid Alkaloids

**DOI:** 10.3389/fmicb.2021.775665

**Published:** 2022-01-05

**Authors:** Shan-Shan Wang, Jia-Meng Liu, Jing Sun, Ya-Tao Huang, Nuo Jin, Min-Min Li, Yan-Tian Liang, Bei Fan, Feng-Zhong Wang

**Affiliations:** Key Laboratory of Agro-Products Quality and Safety Control in Storage and Transport Process, Ministry of Agriculture and Rural Affairs/Institute of Food Science and Technology, Chinese Academy of Agricultural Sciences, Beijing, China

**Keywords:** stems of *Dendrobium*, endophytic bacteria, Illumina MiSeq sequencing, antimicrobial activity, dendrobine-type sesquiterpenoid alkaloids, biosynthetic pathway of dendrobine, whole endophytic bacterial genome analysis

## Abstract

As the unique component of *Dendrobium*, dendrobine-type sesquiterpenoid alkaloids (DSAs) possess a variety of medicinal properties. It has been well documented that plant endophytes can *in vitro* synthesize secondary metabolites identical or similar to metabolites produced by their host plants. This study aimed to investigate the composition and distribution of endophytic bacteria of *Dendrobium* stems by Illumina MiSeq platform sequencing and cultivation-dependent methods and then to assess the potential for endophytic bacteria to produce DSAs. Results indicated that it was necessary to combine both cultivation-dependent and cultivation-independent methods to analyze the community structure of endophytic bacterial in plants comprehensively. The length of the *Dendrobium* stems influenced the endophytic bacterial community. The diversity and richness of endophytic bacteria in group J10_15cm of stems were the highest, which showed a significant difference from the other stem groups. However, there was no certain connection between the diversity and richness of endophytic bacteria and the content of dendrobine. It was most likely due to the influence of several specific endophytic bacteria genera, such as *Sphingomonas* and *Rhodococcus*. *Athelia rolfsii*, *Myrothecium roridum*, as pathogenic fungi, and *Pectobacterium carotovorum* subsp. *actinidiae*, as pathogenic bacteria of *Dendrobium*, were used to determine the antimicrobial activities. In these assays, six strains belonging to five genera showed antimicrobial activity against at least two phytopathogens. The strain BL-YJ10_15-29 (*Paracoccus pueri* THG-N2.35, 98.98%) showed the best antimicrobial activity against the three phytopathogens. In addition, 2 DSAs (6-hydroxydendrobine and nobilonine) were identified in the fermentation supernatant of the strain CM-YJ10_15-44 (*Pseudomonas protegens* CHA0, 99.24%), whereas the whole-genome analysis results further demonstrated that the precursors of the two DSAs [geranyl-PP and (E, E)-famesyl-PP] were synthesized mainly through the methyl-D-erythritol 4-phosphate pathway in this strain. This study provides new insight into the studies on the biosynthesis of DSAs and provides potential biocontrol bacteria.

## Introduction

As a perennial herb in the family Orchidaceae (*Dendrobium* Sw.), *Dendrobium* is widely distributed in the southern parts of Asia, Oceania, and other tropical and subtropical areas. According to The Plants of the World (2021), there are 1,583 species of *Dendrobium* in the world, and more than 80 species have been reported in China, most of which are mainly distributed in the south of Qinling Mountains (Zheng et al., 2018; Wang, 2021). Many species of this genus have been used as traditional Chinese medicine, such as *Dendrobium nobile* Lindl., *Dendrobium huoshanense* C. Z. Tang et S. J. Cheng, and *Dendrobium officinale* Kimura et Migo (Chinese Pharmacopoeia, 2020). Modern medical research has shown that *Dendrobium* possesses various medicinal efficacy, such as antioxidant, antitumor, anti-inflammatory, hypoglycemic, immunity enhancement, and neuroprotective (Tang et al., 2017). The medicinal ingredients mainly consist of polysaccharides, alkaloids, flavonoids, amino acids, bibenzyl, and several trace mineral elements (Zhang et al., 2018; Liu G. Y. et al., 2020; Wang Z. C. et al., 2020). *Dendrobium* was susceptible to many phytopathogens; *Athelia rolfsii* (CBS 719.83), *Myrothecium roridum* (CBS 357.89), and *Pectobacterium carotovorum* subsp. *actinidiae* (BCCM/LMG 26003) are common pathogens of *Dendrobium*, causing southern blight, tar spot, and soft rot disease, respectively (Li H. M. et al., 2017). At present, the main methods of preventing *Dendrobium* diseases are mainly applying chemical pesticides and controlling growth conditions (Jiao et al., 2021). However, there are obvious deficiencies in both two methods. Chemical pesticides are proved to be not safe for humans and the environment, and the method of controlling growth conditions is laborious and time-consuming. Therefore, it is particularly important to find a safer strategy to prevent *Dendrobium* diseases.

As the unique component of *Dendrobium*, 26 dendrobine-type sesquiterpenoid alkaloids (DSAs) have been isolated and identified from *Dendrobium* to date (Li et al., 2019; Liu J. M. et al., 2020; Yin et al., 2020). DSAs show significant activity in protecting hepatic injury, improving brain function, protecting acute cerebral ischemia injury, antitumor, anti-cataract, and anti-influenza A virus (Wang and Ren, 2015; Li R. C. et al., 2017; Li et al., 2019; Ci et al., 2020). Li L. S. et al. (2017) found that DSAs can penetrate the blood–brain barrier and showed neuropharmacological effects such as anti-Alzheimer’s disease and protected neurons from damage. Reports also showed that DSAs could treat influenza virus infection by inhibiting early steps in the viral replication cycle and could be developed as a promising agent (Li R. C. et al., 2017). Therefore, the DSAs show unique advantages and broad development prospects in the development of novel drugs and functional foods. Currently, DSAs are mainly obtained by conventional plant extraction and chemical synthesis, which are limited by the harsh growth conditions of *Dendrobium*, low content of DSAs, long artificial cultivation period, and difficult chemical synthesis. Therefore, it is of great scientific and economic significance to explore novel ways of synthesizing DSAs.

It has been stated that the bioactive secondary metabolites of medicinal plants are commonly associated with their endophytes, which can synthesize secondary metabolites similar or identical to metabolites produced by their host plants (Köberl et al., 2013; Sharma et al., 2021). Li Q. et al. (2017) found that fungus MF23 (*Mycena* sp.) could increase the content of dendrobine in host *Dendrobium* by regulating the expressions of genes involved in the mevalonate (MVA) pathway, which related to the sesquiterpene skeleton formation of DSAs. Sarsaiya et al. (2020) detected the presence of dendrobine in the *Trichoderma longibrachiatum* MD33, which was isolated from *D. nobile* stems. Endophytes could produce a large amount of novel and bioactive secondary metabolites that are not only beneficial to the host plants but also economically important to humans for the potential applications in pharmaceutical, agricultural, and food industries (Gutierrez et al., 2012). Studies have shown that the community structure of endophytes is affected by host plant and multiple environmental factors (Wu et al., 2021). Host plants can also influence their microbiome by secreting various metabolites in plant tissue (Pang et al., 2021). Studies have demonstrated that the content and composition of secondary metabolites in *Dendrobium* stems of different lengths are different (Huang et al., 2021). However, to the best of our knowledge, no one has yet investigated the community structure of endophytic bacterial of *Dendrobium* stems with different lengths.

In the published literature, most of the studies focused on endophytic fungi of *Dendrobium*; only the ability of endophytic fungi of *Dendrobium* to produce DSAs has been studied and confirmed (Sarsaiya et al., 2020). However, a small number of studies have focused on endophytic bacteria (Harrison and Griffin, 2020). In particular, the studies of the bioactive secondary metabolites of endophytic bacteria are rare. The present study is the first attempt to comprehensively study the community structure of endophytic bacterial of *Dendrobium* stems with different lengths by Illumina MiSeq platform sequencing and cultivation-dependent methods. Meanwhile, the potential ability of endophytic bacteria to produce DSAs was investigated by ultra-high performance liquid chromatography/triple quadrupole mass spectrometry (UHPLC-QqQ-MS/MS) analysis, whole-genome sequencing, and ultra-high performance liquid chromatography/quadrupole time-of-flight mass spectrometry (UHPLC-Q-Tof-MS^E^) analysis. In addition, the Kirby–Bauer test was used to determine the antimicrobial activity of the metabolites of isolated endophytic bacterial strains against pathogens. The purpose of this study was to understand better the interactions between the endophytic microbial community and *Dendrobium* and to obtain endophytic bacterial strains with biocontrol activity or DSA-producing ability. This study also aims to preliminarily explore the biosynthetic pathway of DSAs and establish a foundation for the biosynthesis of DSAs.

## Materials and Methods

### Sample Collection

*Dendrobium* stems, which grew on the rock wall, were collected from Sanming City, Fujian Province, China, in April 2019 (latitude 25°29′–27°07′, longitude 116°22′–118°39′, temperature 18–22°C) and labeled according to the difference of the length as group J0_5cm (the length of the stem is from 0 to 5 cm), group J5_10cm (5–10 cm), group J10_15cm (10–15 cm), and group J15cm (longer than 15 cm). Each group was subdivided into three replicates. All samples were authenticated as the stems of *Dendrobium* by Professor Pengfei Tu. A portion of these stem samples was surface-sterilized as the method in Wang et al. (2019) to avoid the influence of microorganisms on the surface (Supplementary Figure 1), and the rest were determined for dendrobine content.

### Ultra-High Performance Liquid Chromatography/Triple Quadrupole Mass Spectrometry Analysis of Dendrobine-Type Sesquiterpenoid Alkaloids

The stem samples were freeze-dried with a freeze dryer (FD-1C Freezing Dryer, Detianyou Technology Development Co., Ltd., Beijing, China) and pulverized mechanically with a high-speed pulverizer (XuMan, Bo Ou Hardware products Co., Ltd., Yongkang, China). Then, the powders were sieved through a 40-mesh sieve before starting the experiment. Dendrobine was extracted according to the method in Chinese Pharmacopoeia (version 2020) with modifications (Chinese Pharmacopoeia, 2020). In brief, 0.25-g stem powder was added into a 25-ml methanol solution containing 0.05% formic acid. The mixture was ultrasound for 30 min and refluxed at 75°C for 3 h to extract as much dendrobine as possible. After extraction, the weight loss was complemented with the solvent. The supernatant was blown with nitrogen to dry, and the residue was dissolved in 1-ml chromatographic pure methanol and passed through a 0.22-μm filter membrane for UHPLC-QqQ-MS/MS analysis (Supplementary Figure 2). The stock solutions of dendrobine (1.0 mg/ml) were prepared in chromatographic pure methanol. Calibration plots were offset at 0.03125, 0.0625, 0.125, 0.25, 0.5, 1, and 2 ng/ml. A liquid chromatography–tandem mass spectrometry (LC-MS/MS) system with Agilent 1,290 Infinity II and Agilent 6,470 LC/TQ (Agilent Technologies Inc., Santa Clara, CA, United States) equipped with an electrospray ionization (ESI) interface was used. An InfinityLab Poroshell 120 EC-C18 (2.1 × 50 mm, 2.7 mm) column was used for UHPLC separations. The autosampler and the column temperature were kept at 4 and 35°C, respectively. Solvent A (0.1% formic acid) and solvent B (acetonitrile) were used as mobile phases at a flow rate of 0.3 ml/min. The gradient elutions were as follows: 15% B (0–0.5 min), 15–45% B (0.5–2.0 min), 45–99% B (2.0–2.5 min), 99% B (2.5–3.5 min), 99–15% B (3.5–3.6 min), and 15% B (3.6–4.0 min). The injection volume was 2 μl. Multiple reactions monitoring mode was used in the positive ion mode. The MS parameters were as follows: cell accelerator voltage 3.5 kV, dwell 80, and fragmentor 130. The qualifying and quantifying ions (m/z) for dendrobine were 264.2→70.0 (m/z) (collision energy: 42) and 264.2→108.0 (m/z) (collision energy: 40), respectively. All of the data were acquired using Agilent MassHunter Workstation software (LC/MS Data Acquisition) version 10.0 and processed using Agilent MassHunter Workstation software (qualitative analysis) version 10.0.

### Illumina MiSeq Sequencing and Data Analysis

Microbial DNA was extracted from the surface-sterilized stems using the FastDNA SPIN kit (MP Biomedicals, Solon, United States) (Hu et al., 2020). The V5–V7 region of the bacteria 16S rRNA gene sequences was amplified by polymerase chain reaction using primers 799f (5′-AACMGGATTAGATACCCKG-3′) and 1193r (5′-ACGTCATCCCCACCTTCC-3′) (Wang et al., 2019). Amplicons were extracted from 2% agarose gels and purified using the AxyPrep DNA Gel Extraction Kit (Axygen Biosciences, Union City, CA, United States); then, it was quantified using QuantiFluor*™*-ST (Promega, United States). Purified amplicons were pooled in equimolar and paired-end sequenced (2 × 300) on an Illumina MiSeq platform (Illumina, San Diego, United States) by Shanghai Majorbio Bio-pharm Technology Co., Ltd. The raw reads were stored in the Sequence Read Archive of the National Center for Biotechnology Information (NCBI) database. Sequencing data were processed and analyzed according to the method in Liu J. M. et al. (2020) (Supplementary Figure 3).

### Isolation and Identification of Culturable Endophytic Bacteria

All the four groups of surface-sterilized stems were cut into 0.5–1.0-cm segments by sterile surgical scissors and placed onto 11 different media added with 1% plant extracts of stems (Wang et al., 2019; Supplementary Table 1). Six to eight 0.5–1.0-cm segments were placed on each medium. They were then incubated at 28°C, and the bacterial colonies were isolated and purified once they emerged. The emergence of new bacterial colonies was checked every day until there were no new colonies for approximately a month. The single colony was purified at least three times on the YIM38 agar medium. Selected isolates were long term stored at −80°C using 50% (v/v) glycerol [bacterial fluid–glycerol (50%) = 1:1, v/v]. The criterion for strain nomenclature was as follows: type of medium–YJ stem length–strain number. YJ means that endophytes were isolated from stems of *D. officinale*. For example, CM-YJ10_15-44 indicates the 44th strain isolated from group J10_15cm (the length of the stem is from 10 to 15 cm) of *D. officinale* in the CM medium.

### Screening for Antimicrobial Activity of Culturable Endophytic Bacteria

The antimicrobial activities of the culturable endophytic bacteria against phytopathogens *A. rolfsii* (CBS 719.83), *M. roridum* (CBS 357.89), and *P. carotovorum* subsp. *actinidiae* (BCCM/LMG 26003) were investigated. The solvent extract of supernatant and sediment of endophytic bacterial isolates were collected and redissolved in 1 ml of methanol separately according to the method by Wang et al. (2019) (Supplementary Figure 4). The *in vitro* antibacterial activity of these strains was carried out by the Kirby–Bauer test (Barry et al., 1979). Methanol was used as a negative control. The diameter of the inhibition zones was measured using an electronic digital caliper (0–150 mm) after the plates were incubated for 16 h at 28°C under aerobic conditions.

### Whole-Genome Sequencing and Data Analysis of *Pseudomonas protegens*

The strain CM-YJ10_15-44 in the mid-log phase (8 h) was collected and sent to the Shanghai Majorbio Bio-pharm Technology Co., Ltd. for whole-genome sequencing. The reasons and procedures for selecting this strain are shown in Supplementary Figure 5. Genomic DNA was extracted using Wizard^®^ Genomic DNA Purification Kit (Promega), and the purified genomic DNA was quantified by TBS-380 fluorometer (Turner BioSystems Inc., Sunnyvale, CA, United States). High-quality DNA (OD260/280 = 1.8–2.0, > 20 μg) was used for further research. The whole genome was sequenced using a combination of PacBio RS II Single-Molecule Real-Time and Illumina sequencing platforms. For Illumina sequencing, the genomic DNA library was constructed by using the NEXTflex*™* Rapid DNA-Seq Kit for sequencing on Illumina HiSeq X Ten machine according to the method by Zhu et al. (2020). Pacific Biosciences sequencing was carried out according to the method by Si et al. (2019). Bioinformatics analysis of data generated by PacBio and Illumina platforms was performed using the I-Sanger Cloud Platform^1^. The complete genome sequence was assembled using both the PacBio reads and Illumina reads according to the method by Zheng et al. (2017). Glimmer version 3.02^2^ was used for the identification of predicted coding sequences (CDS). Barrnap version 0.8^3^ and tRNA-scan-SE version 2.0^4^ were used for rRNA and tRNA prediction, respectively. The predicted CDSs were annotated by comparison with the Kyoto Encyclopedia of Genes and Genomes database^5^.

### Ultra-High Performance Liquid Chromatography/Quadrupole Time-of-Flight Mass Spectrometry Analysis of Dendrobine-Type Sesquiterpenoid Alkaloids

Ultra-high-performance liquid chromatography (Waters ACQUITY) and quadrupole time-of-flight mass spectrometry (Waters Xevo G2QTOF) were used to analyze metabolites in the solvent extracts from the fermentation supernatant of strain CM-YJ10_15-44. YIM38 medium was used for fermentation. The separation was carried out on a Waters ACQUITY UHPLC^R^ HSS T3 column (100 × 2.1 mm, 1.8 μm) equipped with a guard column (ACQUITY UHPLC^R^ HSS T3 1.8 μm). The autosampler and the column temperature were kept at 4 and 35°C, respectively. Solvent A (0.1% formic acid) and solvent B (acetonitrile) were used as mobile phases at a flow rate of 0.3 ml/min. The gradient elutions were as follows: 15–40% B (0–3 min), 40–85% B (3–8 min), 85–100% B (8–10 min), 100% B (10–12 min), 100–15% B (12–12.5 min), and 15% B (12.5–15 min). The injection volume was 3 μl.

Waters Xevo G2QTOF mass spectrometer connected to the UHPLC system *via* an ESI interface was used for high-resolution ESI−MS experiments. The ESI source was operated in the sensitivity positive ion mode with the capillary voltage at 3.0 kV. The source and desolvation temperatures were 100 and 280°C, respectively. The cone and desolvation gas flows were 30 and 600 L/h, respectively. The sample and extraction cones were 40 and 4 V, respectively. All data were collected by MassLynx software (version 4.1). The data were acquired using an MS^E^ method with an m/z range of 50–1,200 (Wang et al., 2016). Data processing was performed using UNIFI (version 1.9.4.053, Waters, Milford, MA, United States) (Zuo et al., 2019). Key parameters of UNIFI were depicted as follows. The selected libraries: Waters Traditional Medicine Library and traditional Chinese medicines—*Dendrobium*; selected positive adducts: + H, + K, + Na, and + NH_4_; reference mass: 556.2766 m/z; mass error: −10 to 10 ppm. Flow diagram of UHPLC-Q-Tof-MS^E^ analysis has been provided as Supplementary Figure 6.

## Results

### Ultra-High Performance Liquid Chromatography/Triple Quadrupole Mass Spectrometry Analysis of Dendrobine Compounds

**FIGURE 1:**
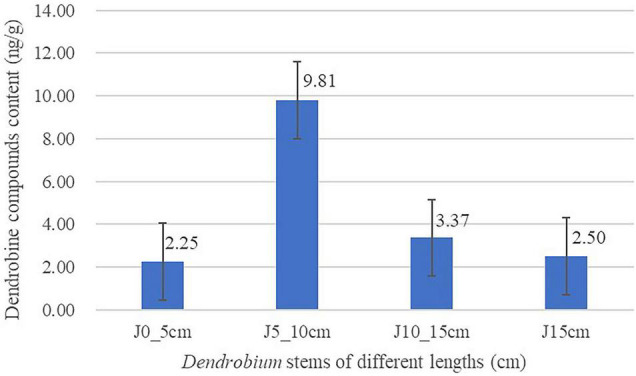
Content of dendrobine in *Dendrobium* stems of different lengths. Three replicates for each condition. Error bars represent standard error (*n* = 3).

Linear regressions of the responses of peak area and concentrations were fitted over the concentration range 0.03125–2 ng/ml for dendrobine. The calibration curve was Y = 6652.2 * X + 149.41 (*R*^2^ = 0.9964). The limit of quantitation and limit of detection for the determination of dendrobine were 0.05 and 0.03 ng/ml, which is defined as a signal/noise ratio of 10 and 3, respectively. It was used to detect the content of dendrobine in *Dendrobium* stems of different lengths. The result is shown in Figure 1. Dendrobine content of group J5_10cm was highest (9.81 ng/g), followed by group J10_15cm (3.37 ng/g), and group J0_5cm was the lowest one (2.25 ng/g).

### Illumina MiSeq Sequencing and Data Analysis

#### Quality Control and Analysis of Sequencing Data

After clustering at 97% similarity level, a total of 483,572 sequences and 2,862 operational taxonomic units (OTUs) were obtained from the four groups. Three parallels in each group. It indicated that the sequencing data were large enough to reflect the vast majority of microbial diversity information, which can be seen from the gradual flattening of the rarefaction curves of the Shannon diversity index (Supplementary Figure 7). In other words, the rarefaction curves showed that the sequencing work was relatively comprehensive in covering the bacterial diversity, as the curves tended to approach saturation, indicating that the selected sequence data adequately reflected the bacterial abundance of these samples. The alpha-diversity analyses, including Shannon, Sobs, Simpson, Chao, Ace, and Coverage, were used to detect the distribution of endophytic microbial composition on OTU level and estimated the endophytic community complexity (Liu J. M. et al., 2020; Supplementary Table 2). The lower Simpson index value but higher Shannon index of group J10_15cm indicated greater community diversity. The higher Chao index indicated a higher richness of endophytic bacterial community in group J10_15cm.

**FIGURE 2:**
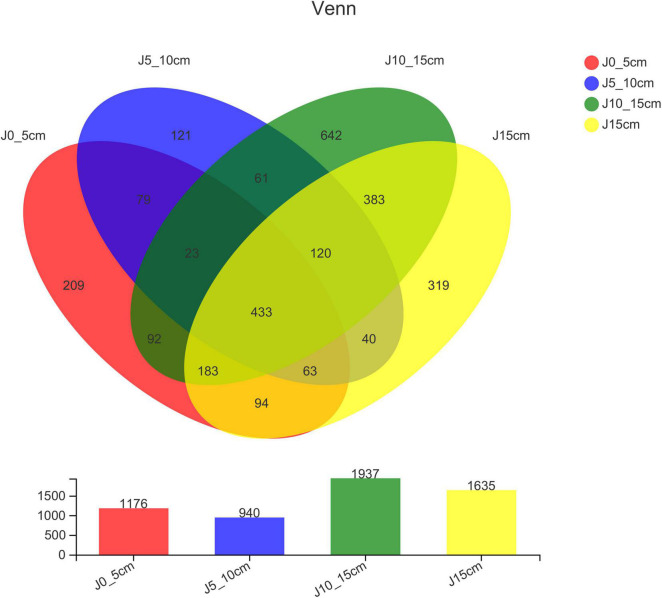
Venn diagrams showing number of OTUs shared and unique among different samples (group J0_5cm represents length of stems was from 0 to 5 cm; group J5_10cm represents length of stems was from 5 to 10 cm; group J10_15cm represents length of stems was from 10 to 15 cm; group J15cm represents length of stems was longer than 15 cm).

The differences in OTU abundance in the four groups were shown in the Venn diagram to indicate the similarity and overlap of species composition in different samples intuitively. Group J10_15cm showed the highest OTU abundance of endophytic bacterial, whereas group J5_10cm was the lowest. All the four groups shared 433 same OTUs, whereas harbored 209 (group J0_5cm), 121 (group J5_10cm), 642 (group J10_15cm), and 319 (group J15_20cm) unique OTUs, respectively (Figure 2).

#### Composition and Diversity of Endophytic Bacterial Community in Different Groups

**FIGURE 3:**
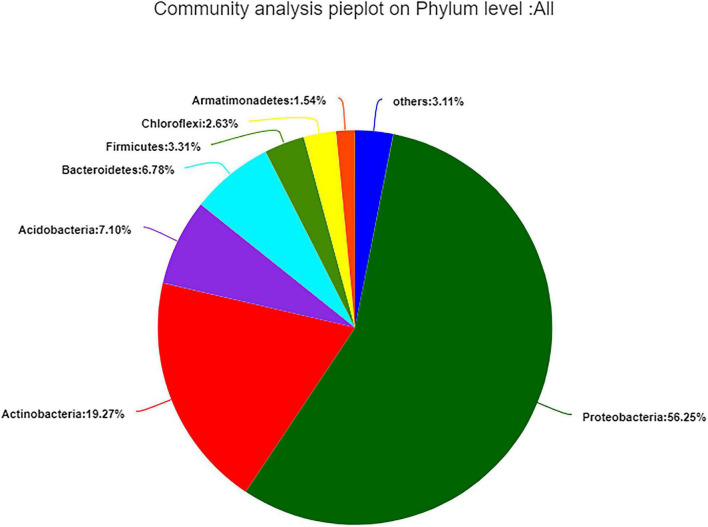
Composition and relative abundance of endophytic bacterial in different samples on phylum level. Phyla making up less than 1% of total composition in samples were classified as “other.”

The 99.99% of bacterial sequences were classified into 33 different phyla, 68 classes, 183 orders, 366 families, 766 genera, and 1,313 species, whereas 0.01% were unclassified. *Proteobacteria* (56.25%) was the dominant phylum, followed by *Actinobacteria* (19.27%); the remaining 3.11% contained 26 low-abundant phyla (Figure 3).

**FIGURE 4:**
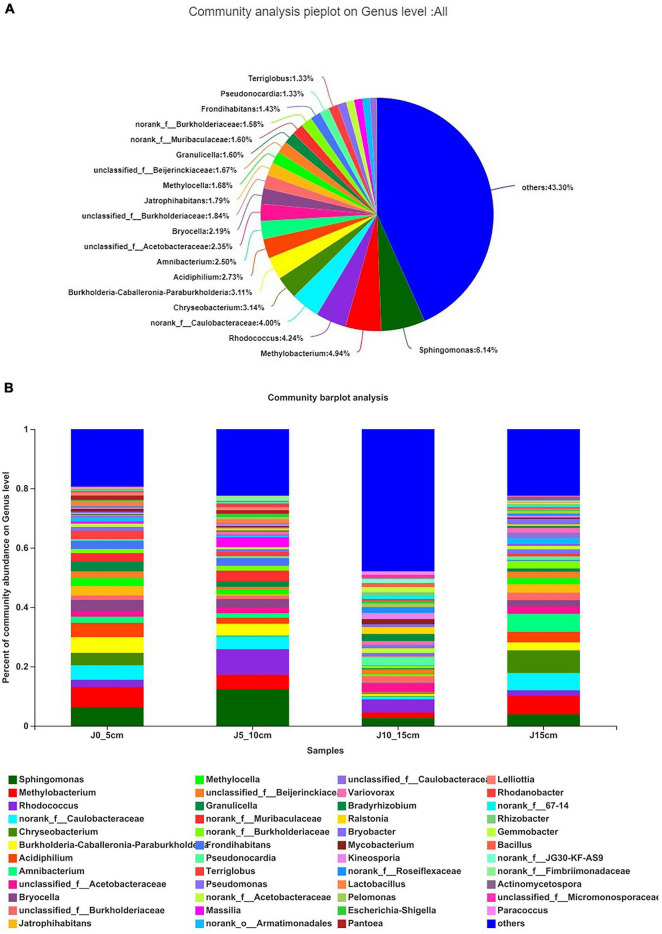
Composition and relative abundance of endophytic bacterial on genus level. **(A)** Community analysis pieplot on genus level of culture-independent endophytic bacteria on whole. **(B)** Composition and relative abundance of endophytic bacterial in different samples on genus level. Color of column represents different genera, and length of column represents proportion size of genus. Sequences that could not be classified into any known group were assigned as “unclassified.” Genera making up less than 1% of total composition in each sample were classified as “other” (group J0_5cm represents length of stems was from 0 to 5 cm; group J5_10cm represents length of stems was from 5 to 10 cm; group J10_15cm represents length of stems was from 10 to 15 cm; group J15cm represents length of stems was longer than 15 cm).

The results showed that the distribution of endophytic bacterial was different in different lengths of *Dendrobium* stems. Overall, *Sphingomonas* (6.14%) accounted for the highest proportion, followed by *Methylobacterium* (4.94%) and *Rhodococcus* (4.24%), whereas there were 43.30% genera with below 1% abundance (Figure 4A). The community barplot analysis indicated that the bacterial richness of the group J10_15cm was the highest. The high proportion of “others” indicated that there were more endophytic bacteria genera with an abundance of less than 1% in this group, so it possessed a higher bacterial richness than the other groups (Figure 4B). The dominant genera of each group are shown in Supplementary Table 3. There were 19 genera with over 1% abundance in group J0_5cm, whereas the dominant genera were *Methlobacterium* (6.85%) and *Sphingomonas* (6.22%). There were 18 genera with over 1% abundance in group J5_10cm, whereas the dominant genera were *Sphingomonas* (12.39%) and *Rhodococcus* (8.63%). There were 17 genera with over 1% abundance in group J10_15cm, whereas the dominant genera were *Rhodococcus* (4.39%) and *Pseudonocardia* (2.95%). There were 19 genera with over 1% abundance in group J15cm, whereas the dominant genera were *Chryseobacterium* (7.65%) and *Methylobacterium* (6.24%). There were significant differences in the abundance of the same genus among the four groups. For instance, *Rhodococcus* (8.63%) and *Sphingomonas* (12.39%) showed a higher proportion in group J5_10cm than the other three groups. *Bacillus* (1.33%), *Bradyrhizobium* (2.44%), *Gemmobacter* (1.75%), *Kineosporia* (2.05%), *Mycobacterium* (1.73%), *Paracoccus* (1.16%), *Pseudonocardia* (2.95%), and *Ralstonia* (2.29%) were only present at abundance levels greater than 1% in group J10_15cm (Supplementary Table 3).

**FIGURE 5:**
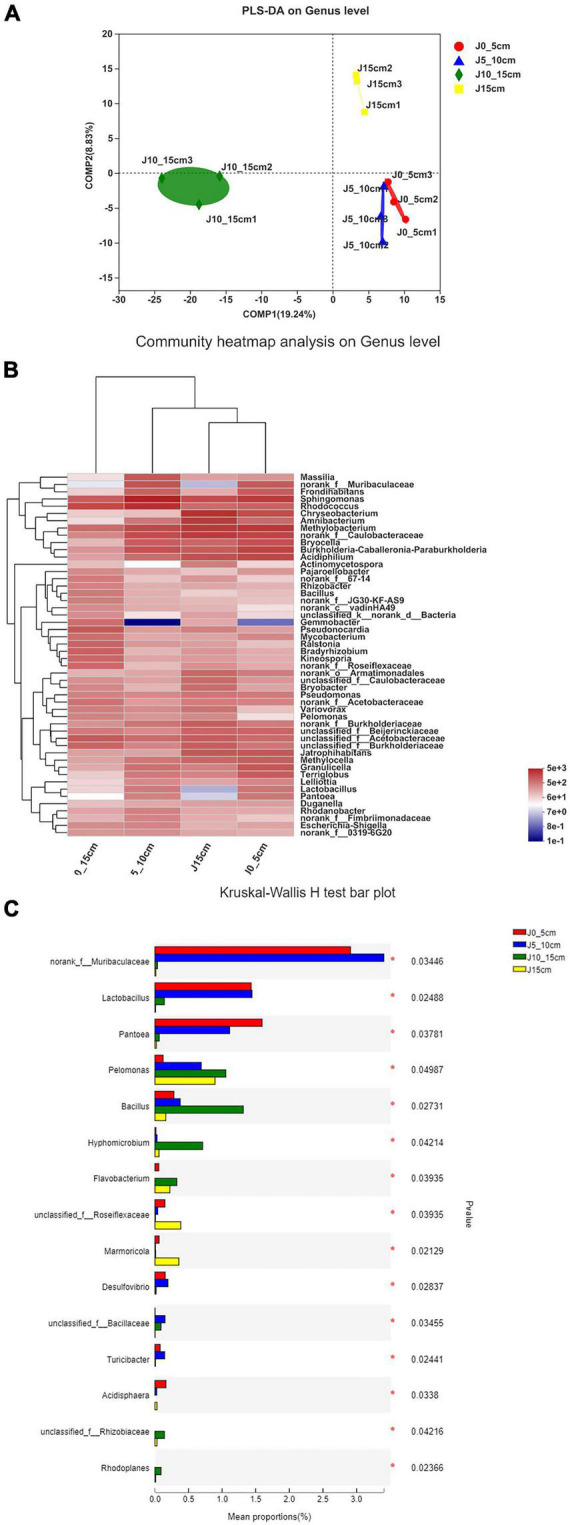
Differences among samples in different samples on genus level. **(A)** Partial least squares discriminant analysis illustrates differences between bacterial communities in four groups. **(B)** Heatmap of top 50 most abundant genera in bacterial communities detected in four groups. Dendrograms for hierarchical cluster analysis grouping genera and sample locations were shown at left and top, respectively. **(C)** Kruskal–Wallis *H*-test bar plot of genera with a high relative abundance and statistically significant differences. *0.01 < *P* ≤ 0.05 (group J0_5cm represents length of stems was from 0 to 5 cm; group J5_10cm represents length of stems was from 5 to 10 cm; group J10_15cm represents length of stems was from 10 to 15 cm; group J15 cm represents length of stems was longer than 15 cm).

The results of partial least squares discriminant analysis showed significant differences among different groups at the genus level. Except for the length of the stems, all other experimental conditions were kept identical among the different *Dendrobium* samples, which indicated a significant relationship between the length of the *Dendrobium* stems and the community composition of endophytic bacteria (Figure 5A). As shown in the heatmap of hierarchical clustering of endophytic bacterial communities, the group J10_15cm was significantly different from the other groups (Figure 5B). Kruskal–Wallis *H*-test was used to analyze the differences between the groups. Only genera with a high relative abundance and statistically significant differences were plotted. As shown in Figure 5C, the relative abundances of *norank_f_Muribaculaceae*, *Desulfovibrio*, *unclassified_f_Bacillaceae*, and *Turicibacter* in group J5_10cm were significantly higher than other groups, and the relative abundances of *Pelomonas*, *Bacillus*, *Hyphomicrobium*, *Flavobacterium*, *unclassified_f_Rhizobiaceae*, and *Rhodoplanes* were significantly higher in group J10_15cm than other groups (0.01 < *P* ≤ 0.05).

### Isolation and Identification of Culturable Endophytic Bacteria

A total of 186 cultivable endophytic bacteria isolates were isolated. All the strains were identified by 16S rRNA gene sequence analysis and compared by the EzBioCloud database. The endophytic bacteria were classified into 3 different phyla (*Firmicutes*, *Proteobacteria*, and *Actinobacteria*), 14 genera, and 35 species. The 16S rRNA gene sequences of the 35 species have been submitted to the NCBI GenBank with the accession numbers (MZ674046–MZ674080) and dominated by *Bacillus* (37.14%) and *Lysinibacillus* (11.43%) (Supplementary Figure 8). The evolutionary phylogenetic relationship of 35 isolates was revealed by constructing a phylogenetic tree (Supplementary Figure 9). In genera diversity, *Bacillus* was the dominant one harboring 13 strains, followed by *Lysinibacillus* with four strains. Each of the genera *Curtobacterium*, *Microbacterium*, *Pseudomonas*, *Kosakonia*, *Paracoccus*, and *Staphylococcus* harbored two strains, respectively. Meanwhile, other genera harbored only one strain for each.

### Screening for Antimicrobial Activity of Culturable Endophytic Bacteria

The antimicrobial activity of the 35 strains was tested using the Kirby–Bauer method (Supplementary Table 4). Six strains showed antimicrobial activity against at least two phytopathogens, which belonged to five different genera, namely *Paracoccus*, *Pseudomonas*, *Microbacterium*, *Bacillus subtilis*, and *Streptomyces*. All the six strains showed antimicrobial activity against the *A. rolfsii* and *M. roridum* in this study, whereas only strain BL-YJ10_15-29 and GP-YJ15-74 showed antimicrobial activity against the *P. carotovorum* subsp. *actinidiae* (Table 1). The supernatant showed better antimicrobial activity than sediment overall. The extracts of the strain BL-YJ10_15-29 (*P. pueri* THG-N2.35, 98.98%) showed the best antimicrobial activity against the three phytopathogens.

**TABLE 1:** Endophytic strains that showed antimicrobial activity against at least one of three phytopathogens (diameter of filter paper, 6 mm).

Strain number	GenBank accession number	Closest species in 16S rRNA gene sequences database	Similarity (%)	Active part	Pathogenic fungi		Pathogenic bacteria
					Inhibition rate to *Athelia rolfsii* (mm)	Inhibition rate to *Myrothecium roridum* (mm)	Inhibition rate to *Pectobacterium carotovorum* subsp. *actinidiae* (mm)
BL-YJ10_15-29	MZ674074	*Paracoccus pueri* THG-N2.35	98.98	Supernatant	20.30 ± 0.88	19.81 ± 0.12	28.47 ± 0.98
				Sediment	8.70 ± 0.25	8.89 ± 0.08	11.03 ± 0.14
GP-YJ15-74	MZ674075	*Pseudomonas koreensis* Ps 9-14	98.80	Supernatant	11.83 ± 0.09	14.70 ± 0.63	15.74 ± 0.59
				Sediment	7.72 ± 0.17	7.64 ± 0.21	
CM-YJ10_15-44	MZ674076	*Pseudomonas protegens* CHA0	99.24	Supernatant	7.63 ± 0.06	8.48 ± 0.23	–
				Sediment	–	–	–
TW-YJ10_15-50	MZ674070	*Microbacterium hydrothermale* 0704C9-2	100	Supernatant	7.15 ± 0.16	–	–
				Sediment	7.91 ± 0.13	8.16 ± 0.05	–
GP-YJ10_15-64	MZ674056	*Bacillus subtilis* DSM 10	99.93	Supernatant	12.76 ± 0.18	9.98 ± 0.11	–
				Sediment	–	–	–
HV-YJ15-113	MZ674080	*Streptomyces pratensis* ch24	98.83	Supernatant	8.04 ± 0.14	7.17 ± 0.22	–
				Sediment	–	–	–

### Whole-Genome Sequencing and Data Analysis of *Pseudomonas protegens*

**FIGURE 6:**
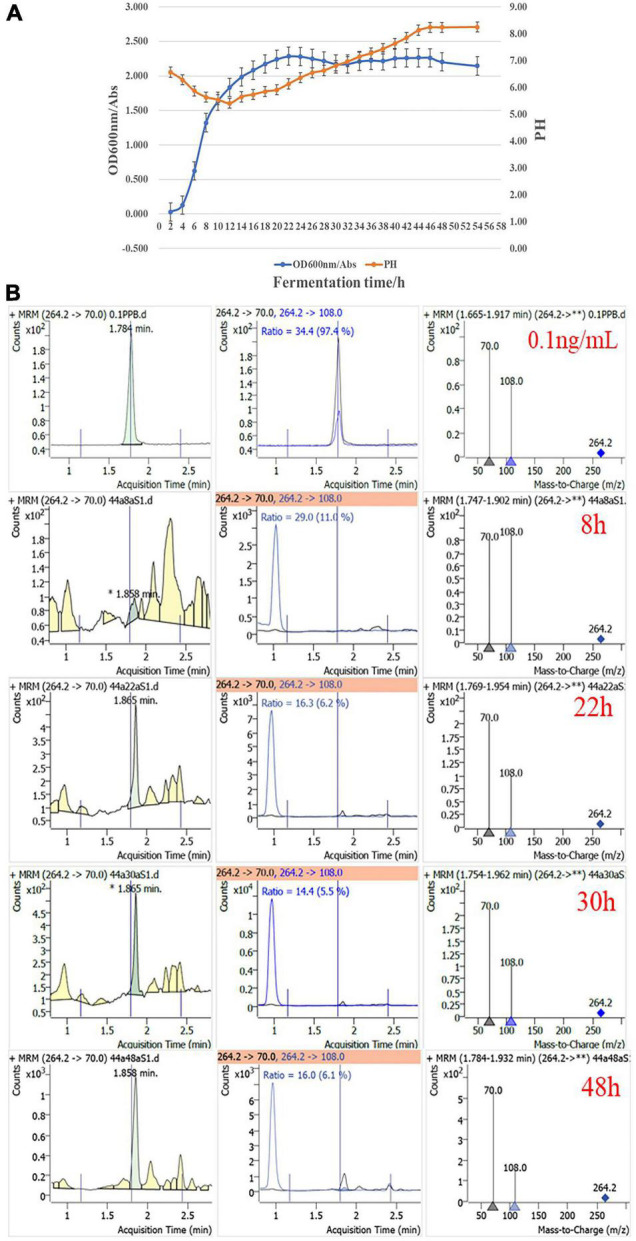
**(A)** Growth curves of strain CM-YJ10_15-44. Error bars represent standard error (*n* = 3). **(B)** UHPLC-QqQ-MS/MS detection results of dendrobine in fermentation supernatant from strain CM-YJ10_15-44 at different time points. 0.1 ng/ml represents concentration of dendrobine standard was 0.1 ng/ml (8 h represents culture supernatant of strain CM-YJ10_15-44 after fermentation for 8 h; 22 h represents culture supernatant after fermentation for 22 h; 30 h represents culture supernatant after fermentation for 30 h; 48 h represents culture supernatant after fermentation for 48 h).

The content of dendrobine in a solvent extract of endophytic bacteria was also determined by UHPLC-QqQ-MS/MS analysis. Dendrobine analogs were detected in a 150-fold concentrated fermentation supernatant of strain CM-YJ10_15-44, isolated from the group J10_15cm. Four time points of the fermentation supernatant were selected, including the mid-log phase (8 h), the end-log phase (22 h), and the stationary phase (30 and 48 h) (Figure 6). Growt8h curves of strain CM-YJ10_15-44 were measured by absorbance at 600 nm with reference to Hod et al. (2010). The results showed that dendrobine analogs were not detected after fermentation for 8 h but were detected after fermentation for 22, 30, and 48 h.

The whole genome of strain CM-YJ10_15-44 was completely sequenced to verify the ability to synthesize DSAs, which showed 99.24% similarity to *P. protegens* strain CHA0 based on the phylogenetic analysis. Whole-genome sequencing of this strain produced a total genome size of 6,953,246 bp and G + C content of 63.36%. A total of 6,387 CDSs were conserved, and 71 tRNA and 16 rRNA were predicted in strains (Supplementary Table 5). The genome sequence was annotated by comparison with the Kyoto Encyclopedia of Genes and Genomes database, and the top four categories were amino acid metabolism, global and overview map, carbohydrate metabolism, and membrane transport (Supplementary Figure 10).

**FIGURE 7:**
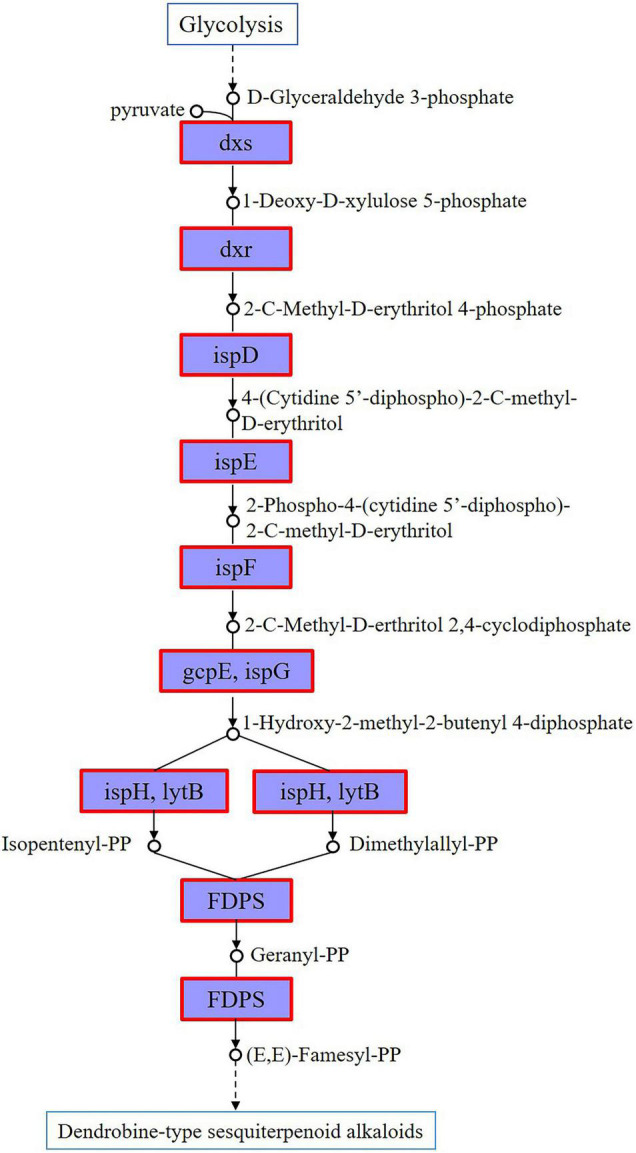
Genes encoding enzymes of MEP pathway annotated in genome of strain CM-YJ10_15-44, which were marked by red borders [dxs represents 1-deoxy-D-xylulose-5-phosphate synthase, chr 6169621–6171522; dxr represents 1-deoxy-D-xylulose-5-phosphate reductoisomerase, chr 1361036–1362226; ispD represents 2-C-methyl-D-erythritol 4-phosphate cytidylyltransferase, chr 1381591–1382286; ispE represents 4-diphosphocytidyl-2-C-methyl-D-erythritol kinase, chr 5820596–5821441; ispF represents 2-C-methyl-D-erythritol 2,4-cyclodiphosphate synthase, chr 1385505–1385978; gcpE, ispG represents (E)-4-hydroxy-3-methylbut-2-enyl-diphosphate synthase, chr 5639742–5640851; ispH, lytB represents 4-hydroxy-3-methylbut-2-en-1-yl diphosphate reductase, chr 5985244–5986191; FDPS represents farnesyl diphosphate synthase, chr 6168621–6169508].

Gene mining on the genes involved in the sesquiterpenoid biosynthesis was conducted on the genome of strain CM-YJ10_15-44. It has been previously shown that the upstream biosynthetic pathway of DSAs is composed of the MVA or methyl-D-erythritol 4-phosphate (MEP) pathway, which provides a basic skeleton for terpenoid alkaloids (Li Q. et al., 2017; Wang Z. C. et al., 2020). In contrast, the downstream post-modification processes of DSAs were unclear. Results in this study showed that all genes encoding enzymes in the MEP pathway had been identified in the strain CM-YJ10_15-44, which was marked by red borders in Figure 7. This suggested that it possessed the biosynthetic capacity of DSAs that were synthesized mainly through the MEP pathway.

In addition, eight secondary metabolite biosynthesis gene clusters with a similarity of more than 20% were identified in strain CM-YJ10_15-44, including T1pks (type I polyketide synthases), T3pks (type III polyketide synthases), Nrps (non-ribosomal peptide), arylpolyene, and other products (Supplementary Table 6). In addition, pyoluteorin, pyrrolnitrin, and diacetylphloroglucinol biosynthesis clusters of this strain have a high homology (100%) to the existing cluster, which suggests that it is most likely to produce these antibiotics.

### Ultra-High Performance Liquid Chromatography/Quadrupole Time-of-Flight Mass Spectrometry Analysis for the Detection of Dendrobine-Type Sesquiterpenoid Alkaloids

UHPLC-Q-Tof-MS^E^ analyses were performed to clearly clarify the DSAs in the strain CM-YJ10_15-44. Two DSAs were identified in the fermentation supernatant of this strain by UNIFI software and related literature. The structural information of the two compounds mainly included the observed retention time (min), mass error (ppm), observed precursor ions (m/z), and high energy fragments (m/z). Mass tolerances for precursor ions were set at ± 10 ppm for precursor ions and ± 5 ppm for high-energy fragments.

Compound 1 produced ions at m/z 318.1472 ([M + K]^+^, C_16_H_25_NO_3_, neutral mass 279.1834), and the characteristic fragment ions at m/z 178.05972 (C_11_H_16_NO^+^) and 207.08641 (C_14_H_25_N^+^) were found in accordance with the fragmentation pattern of dendrobine-type alkaloids. Compound 1 could be tentatively identified as 6-hydroxydendrobine based on the information mentioned earlier. Similarly, compound 2 produced ions at m/z 311.2347 ([M + NH_4_]^+^, C_17_H_27_NO_3_, neutral mass 293.1981), and the characteristic fragment ions at m/z 107.05157 (C_7_H_7_O^+^), 175.04233 (C_13_H_19_^+^), and 221.13803 (C_14_H_21_O_2_^+^), which could be tentatively identified as nobilonine (Wang et al., 2016; Yin et al., 2020; Supplementary Figure 11). 6-Hydroxydendrobine was only detected in the culture supernatant of strain CM-YJ10_15-44 after fermentation for 22 and 30 h; nobilonine was only detected in the culture supernatant of this strain after fermentation for 30 and 48 h. The base peak intensity chromatogram of the fermentation supernatant of this strain CM-YJ10_15-44 at different time points is shown in Supplementary Figure 12.

## Discussion

Previous studies have clearly shown that DSAs possess a variety of medicinal properties (Wang S. S. et al., 2020; Mou et al., 2021). The content of DSAs in *Dendrobium* is affected by several factors, such as *Dendrobium* type, stalk position, cultivation condition, and harvest period. However, no information about the comparison of the dendrobine content of different *Dendrobium* stems is obtained in the current study (Wang S. S. et al., 2020). Stems from the same cluster of *Dendrobium* plants at the same location were collected. The results described in this study demonstrated that dendrobine content varied a lot between different stem lengths of *Dendrobium*, as it showed an increasing trend first and then followed by a decreasing trend while the stem length increased. As shown in Figure 1, the content of dendrobine appears to reach the peak level in group J5_10cm (9.81 ng/g) and then begins to decline. According to the previous reports and the results in this study, we hypothesized that dendrobine might be degraded or synthesized into dendrobine analogs by the action of aminotransferase, transmethylase, and so on over time (Li, 2018; Cai et al., 2019; Lu et al., 2020). However, the specific mechanisms about it remain poorly understood, and further investigations are needed to confirm these speculations.

At present, only 1% of microorganisms can be isolated and identified in the natural environment, whereas there are still a large number of unknown microorganisms that cannot be isolated and identified by culture-dependent approaches due to a number of factors, such as unsuitable temperature, pH, oxygen and nutrient sources, low richness, and lack of signal and chemical stimulus (Xing et al., 2017; Alam et al., 2021). Therefore, high-throughput sequencing of the V5–V7 region of 16S rRNA gene sequence of endophytic bacteria in *Dendrobium* samples was conducted in this study using Illumina Miseq’s high-throughput sequencing platform to comprehensively and deeply explore the distribution and composition of endophytic bacteria. The results showed that the composition and distribution of the endophytic bacterial community were quite different by the length of the *Dendrobium* stems, and the diversity and richness of endophytic bacteria decreased first and then increased obviously. The diversity and richness of endophytic bacteria in group J10_15cm was the highest, and the endophytic community of group J10_15cm showed a significant difference compared with the other groups. Studies have revealed that the endophytes–plant interaction is both controlled by the genes of organisms and the environmental condition. The colonization patterns and translocation of endophytes is a dynamic process (Singh et al., 2017; Wu et al., 2021). After colonizing the rhizosphere, endophytic bacteria usually colonize the host plant endosphere using a series of traits, including motility, attachment, degradation of plant polymer, and evasion of plant defenses. It can then systemically spread and colonize tissues above the ground (Rosenblueth and Martínez-Romero, 2006; Afzal et al., 2019). For instance, bacteria can be found in the aerial parts of the plant 1 day after the plant roots were exposed to them. Guo et al. (2002) inoculated the roots of tomato plants with salmonellae and found the bacteria in the hypocotyls, cotyledons, and stems of tomatoes the next day. Group J0_5cm showed a higher OTU abundance of endophytic bacterial than group J5_10cm, most likely because the group J0_5cm was closer to the root, which indicated that it possessed the advantage of having more contact with the microorganisms in the environment around the root. However, endophytic bacterial can enter the host plant through tissue lesions and plant stomata (Rincón and Neelam, 2021). The result showed that the endophytic bacterial community showed its highest diversity and richness in group J10_15cm, which may be because the endophytic bacterial in group J10_15cm was not only transferred vertically from the roots but also came in partly through the stomata of the stems and leaves. The diversity and richness of endophytic bacteria in group J15cm showed a slightly decreasing trend, which may be caused by the slow vertical migration of endophytic bacteria. In addition, studies have demonstrated that the content and composition of secondary metabolites in *Dendrobium* stems of different lengths are different (Jin et al., 2016; Huang et al., 2021). Plants can influence their microbiome by secreting various metabolites in plant tissue, promoting the growth of endophytic microorganisms that have easy access to nutrients (Pang et al., 2021). The results of dendrobine detection showed that the content of dendrobine in group J5_10cm was the highest, but the diversity and richness of endophytic bacteria were the lowest. It indicated that there was no certain connection between the higher diversity and richness of endophytic bacteria and the higher content of dendrobine, which might be due to the effect of some specific endophytic bacteria genera, such as *Sphingomonas* and *Rhodococcus*. These results provide new insight into the relation between the endophytic bacteria and plant growth characteristics and provide a reference for future research on the mechanisms of complex endophytic microbial community–plant interaction.

Although more rare bacterial genera can be identified and the composition and distribution of endophytic bacteria can be better characterized based on Illumina Miseq’s high-throughput sequencing technology, only isolated and purified strains can be used for further study to elaborate their growth, metabolism, and physiological and biochemical functions. Therefore, how to improve the culture conditions to isolate more culturable endophytic microorganisms is still a priority and necessary idea (Overmann et al., 2017). Eleven different isolation media were used to satisfy the growth and reproduction conditions of different endophytic bacteria. The results of cultivation-dependent showed that *Bacillus* (37.14%) and *Lysinibacillus* (11.43%) accounted for a major proportion, whereas the abundance of *Bacillus* and *Lysinibacillus* were below 1% in the results of Illumina Miseq’s high-throughput sequencing analysis. The possible reason is that *Bacillus* and *Lysinibacillus* are easy to be isolated and identified for the stronger adaptability and can survive in adverse environments for a long time (Radhakrishnan et al., 2017). However, those two genera mostly exist in plants in the form of spores with thick cell walls, which cannot be cleaved better during extraction of plant genome DNA, resulting in the failure to effectively obtain the genomic DNA of those two genera. Thus, it is necessary to combine cultivation-dependent and cultivation-independent analysis to comprehensively analyze the distribution and composition of the endophyte communities in plants. On the other hand, the results of Illumina Miseq’s high-throughput sequencing can be used to analyze the dominant endophytic bacterial in plants and to design specific culture conditions for specific strains so as to improve the methods and conditions for bacterial isolation and culture.

*Dendrobium* was susceptible to many phytopathogens, which have caused serious economic loss to the yield and quality of *Dendrobium*. *A. rolfsii* (CBS 719.83), *M. roridum* (CBS 357.89), and *P. carotovorum* subsp. *actinidiae* (BCCM/LMG 26003) can cause southern blight, tar spot, and soft rot disease, respectively (Koha et al., 2012; Liu et al., 2019; Zhao, 2020). Studies have shown that some endophytes isolated from host plants possessed inhibition activity on phytopathogens (Singh et al., 2017; Carrión et al., 2019). Five different genera of endophytes showed antimicrobial activity against at least two phytopathogens, namely *Paracoccus*, *Pseudomonas*, *Microbacterium*, *B. subtilis*, and *Streptomyces* in this study. The strains BL-YJ10_15-29 (*P. pueri* THG-N2.35, 98.98%) and GP-YJ15-74 (*Pseudomonas koreensis* Ps 9-14, 98.80%) presented better antimicrobial activity. Although there have been many reports on genus *Paracoccus*, most of them have focused on its bioremediate aromatic compounds such as pyrene, halobenzoate, and pyridine; there are few reports about its antimicrobial activity (Aravind et al., 2020; Nakkeeran et al., 2021). Therefore, more attention should be paid to the antibacterial activity of this genus in the future. It has been shown that *Pseudomonas* could benefit their host plants by inducing systemic resistance, promoting growth, suppressing pathogens, and so on (Singh et al., 2017; Chiriboga et al., 2018). In this study, the genus *Pseudomonas* could not only produce DSAs but also enable effective antagonize the phytopathogens of *Dendrobium*. The result of the whole genome of strain CM-YJ10_15-44 revealed that it contained antibiotic biosynthetic pathway gene cluster gene with antimicrobial activity, including pyoluteorin (similarity 100%), pyrrolnitrin (similarity 100%), diacetylphloroglucinol (similarity 100%), orfamide (similarity 94%), and so on, which demonstrated that this strain is more likely to produce these antibiotics. Whether the fermentation broth of strain CM-YJ10_15-44 contains these predicted antibiotics and its antimicrobial activity is related to these antibiotics still needs further in-depth study. This study has provided a solid experimental and theoretical basis for the biological control of *Dendrobium* diseases. In addition, the whole genome analysis results have provided guidance for the study of secondary metabolites with antimicrobial activity.

It has been well documented that plant endophytes can produce similar or identical active secondary metabolites as their host plants (Köberl et al., 2013; Sharma et al., 2021). Since the breakthrough report on the production of taxol by an endophytic fungus of *Taxus brevifolia*, the ability of endophytic microbes to produce metabolites similar to those produced by their host plants has been discovered (Stierle et al., 1993). Endophytes, as a treasure house of bioactive compounds, have received considerable attention. The endophytic *Aspergillus fumigatus* isolated from *Taxus* sp. producing taxol at a concentration of 1.6 g/L (the highest thus far) looks promising for industrial scaleup (Kumar et al., 2019). The results in this study showed that dendrobine analogs were detected in the strain CM-YJ10_15-44. The dendrobine analogs were detected in the culture supernatant after fermentation for 22, 30, and 48 h. Compared with the growth curves, it indicated that dendrobine and its analogs were mainly produced and accumulated in the end-log phase and stationary phase. Studies have shown that the upstream biosynthetic pathway of DSAs is composed of the MVA or MEP pathway, which provides a basic skeleton for sesquiterpenoid alkaloids (Li Q. et al., 2017; Wang Z. C. et al., 2020). The whole-genome analysis results demonstrated that the precursors of the sesquiterpenoid alkaloids pathway [geranyl-PP and (E, E)-famesyl-PP] were synthesized mainly through the MEP pathway in strain CM-YJ10_15-44. 6-Hydroxydendrobine and nobilonine were identified in the fermentation supernatant of this strain by UHPLC-Q-Tof-MS^E^ analysis and UNIFI software. 6-Hydroxydendrobine was detected in the culture supernatant at 22 and 30 h, whereas nobilonine was detected at 30 and 48 h. The results indicated that the presence of DSAs in the fermentation supernatant of this strain was quite dynamic, which might be due to the structural changes under the action of various enzymes. 6-Hydroxydendrobine was an oxygenated derivative of dendrobine, and nobilonine was the product of the cracking of the N-heterocycle ring (A ring) in dendrobine (Okamoto et al., 1966; Wang et al., 2016; Li et al., 2019; Yin et al., 2020). Real-time quantitative analysis of the detected compounds can be carried out to study the rule of dynamic changes in the future. These results provide a strong rationale for the biosynthesis of DSAs. Thus, the content of target products in this strain can be increased through gene regulation or synthetic biology.

## Conclusion

This study revealed a significant relationship between the length of the *Dendrobium* stems and the composition and distribution of the endophytic bacterial community. In contrast, there was no certain connection between the diversity and richness of endophytic bacteria and the content of dendrobine in *Dendrobium* stems. It was most likely due to the effect of specific endophytic bacteria genera such as *Sphingomonas* and *Rhodococcus*. Six endophytic strains with antibacterial activity and one endophytic strain CM-YJ10_15-44 with the ability to produce DSAs were obtained by cultivation-dependent methods. In addition, two DSAs (6-hydroxydendrobine and nobilonine) were identified in the strain CM-YJ10_15-44, and the precursors of the two DSAs were synthesized mainly through the MEP pathway. CM-YJ10_15-44, as a new DSA producer strain, which was discovered in our study, provides new insight into the studies on the biosynthesis of DSAs. In addition, this study further confirmed that plant endophytes could produce similar or identical active secondary metabolites as host plants. However, it is necessary to distinguish that the active secondary metabolites are produced by host plants, by the endophytic microbiome, or by the combination of both in the future, which will dramatically increase the possibilities for exploring its synthesis mechanism. The comprehensive understanding of the community structure of endophytic bacterial in different *Dendrobium* stems by combining the cultivation-dependent and cultivation-independent analysis provided abundant endophytic bacteria resources and laid a foundation for the further study of secondary metabolites and the biocontrol bacteria. The present study provided the bacterial source for plant biocontrol, as it is significant for reducing the use of chemical pesticides, ensuring food safety, and promoting the sustainable development of the *Dendrobium* industry. Meanwhile, this study provided new strategies for the research of DSAs, which can set the stage for DSA biosynthesis and offer an effective way of improving their yield.

## Data Availability Statement

The original contributions generated for this study are publicly available. The 16S rRNA gene sequences of the 35 species have been submitted to the NCBI GenBank with the accession number MZ674046–MZ674080. The Illumina MiSeq sequencing raw data have been submitted to the Sequence Read Archive (SRA) of NCBI with the accession code PRJNA750894. The assembled complete genomes have been submitted to the Genome (WGS) of NCBI with the accession code CP080512.

## Author Contributions

F-ZW, BF, S-SW, and J-ML contributed conception and design of the study. Y-TH, NJ, M-ML, and Y-TL participated in plant collection. S-SW, J-ML, and JS performed the experiments. S-SW and J-ML performed the statistical analysis and were major contributors in writing the manuscript. All authors read and approved the final manuscript.

## Conflict of Interest

The authors declare that the research was conducted in the absence of any commercial or financial relationships that could be construed as a potential conflict of interest.

## Publisher’s Note

All claims expressed in this article are solely those of the authors and do not necessarily represent those of their affiliated organizations, or those of the publisher, the editors and the reviewers. Any product that may be evaluated in this article, or claim that may be made by its manufacturer, is not guaranteed or endorsed by the publisher.

## Footnotes

1
www.i-sanger.com

2
http://ccb.jhu.edu/software/glimmer/index.shtml

3
https://github.com/tseemann/barrnap

4
http://trna.ucsc.edu/software/

5
http://www.genome.jp/kegg/
