# Supplementary material for: Analysis of Endophytic Bacterial Diversity From Different Dendrobium Stems and Discovery of an Endophyte Produced Dendrobine-Type Sesquiterpenoid Alkaloids
Source: Front Microbiol. 2022 Jan 5;12:775665. doi: 10.3389/fmicb.2021.775665 (PMC8767021; doi:10.3389/fmicb.2021.775665)
Supplement: Supplementary file 1 [file Data_Sheet_1.docx]

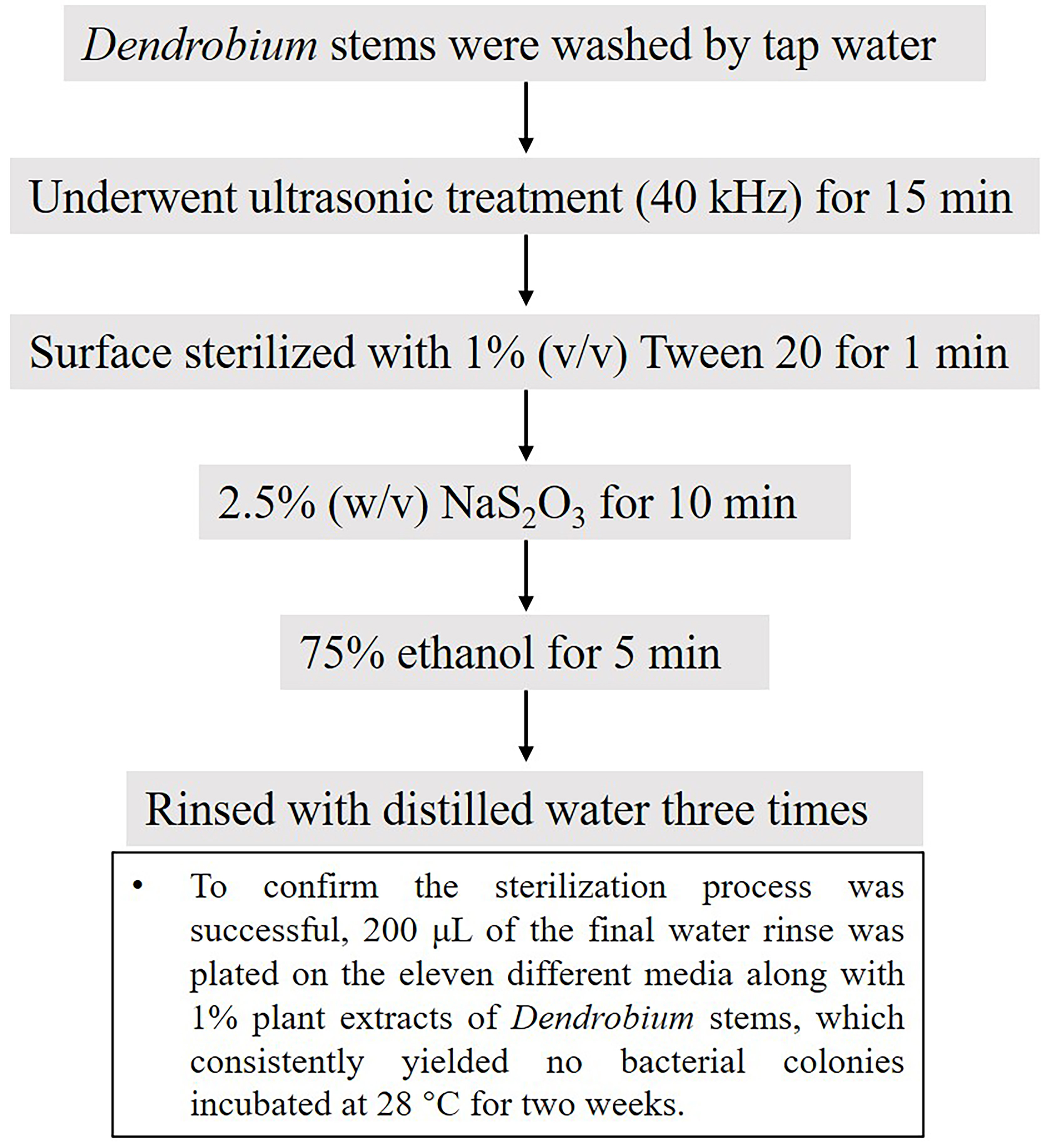


Supplementary Figure 1. Flow diagram of surface sterilization of *Dendrobium* stems.


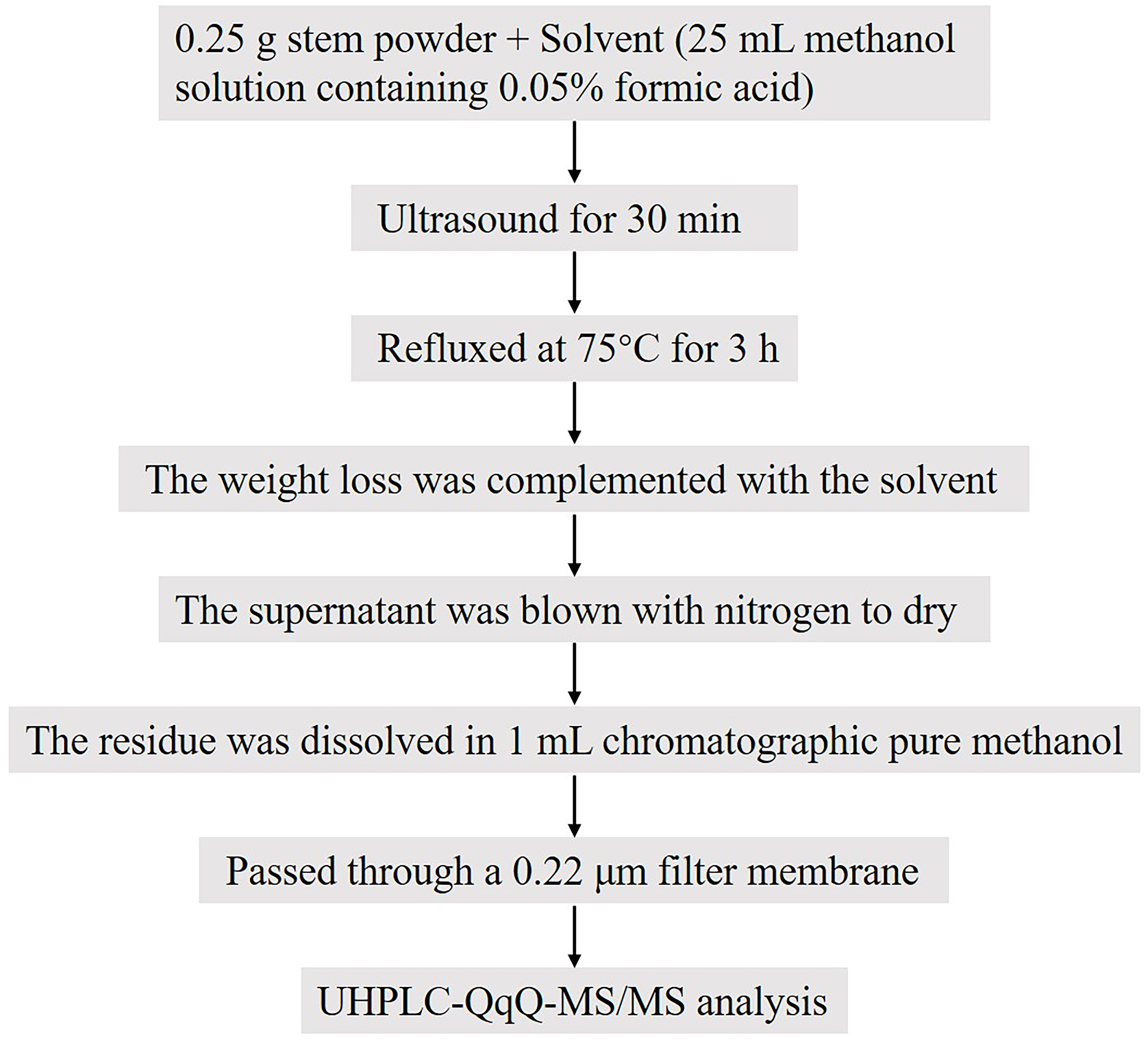


Supplementary Figure 2. Flow diagram of extraction method of dendrobine.


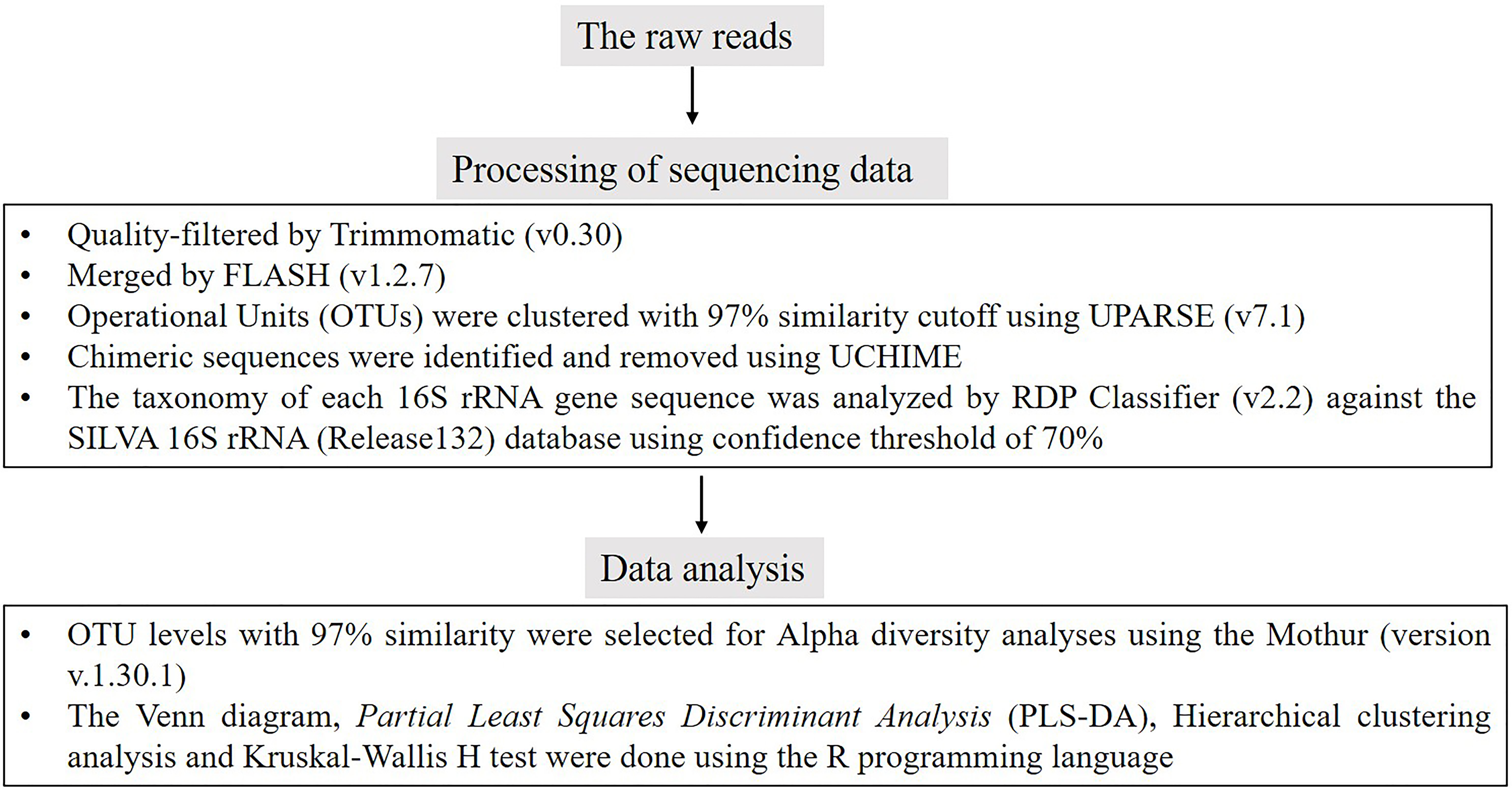


Supplementary Figure 3. Flow diagram of Illumina MiSeq sequencing data processing and analysis.


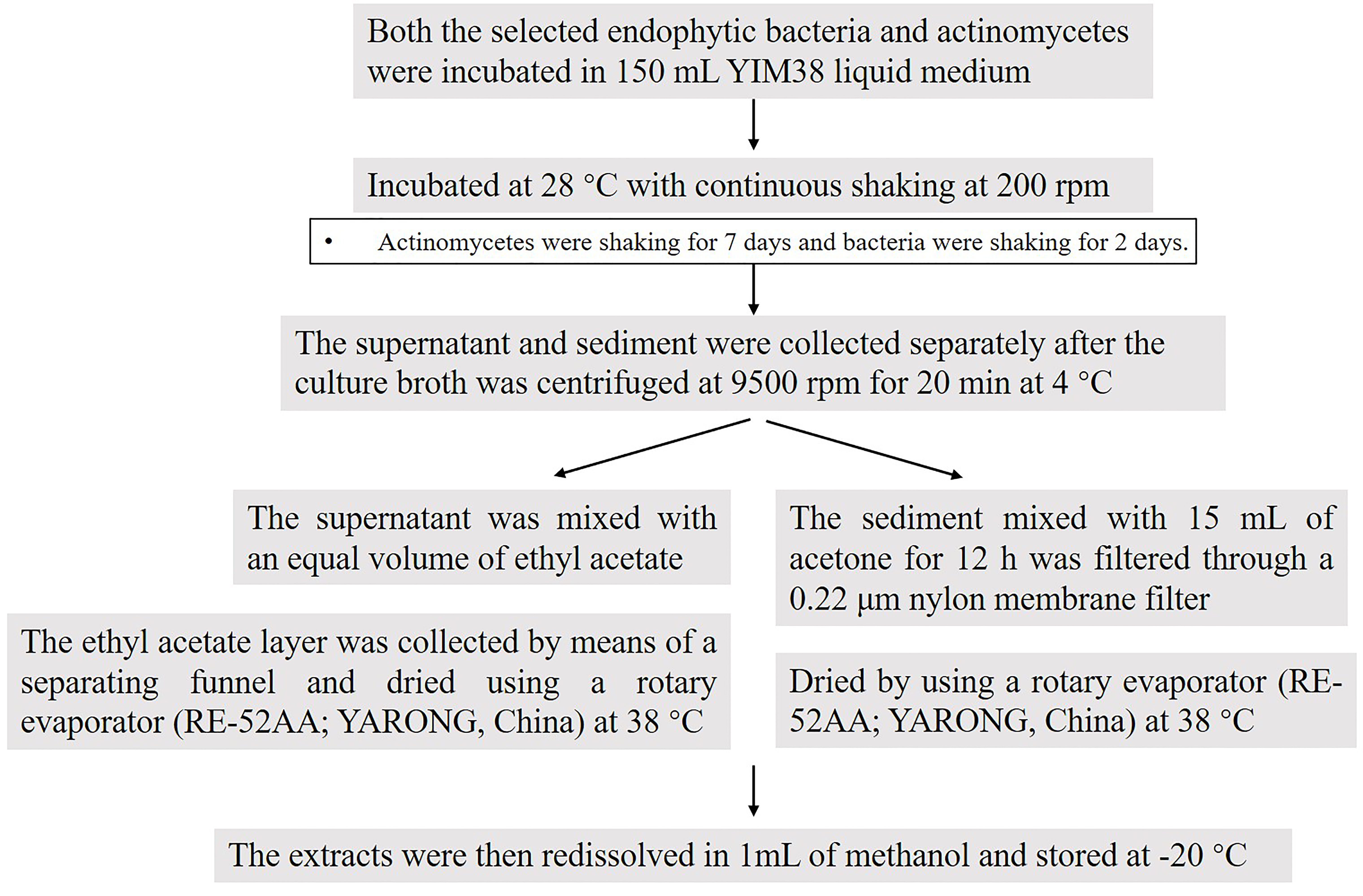


Supplementary Figure 4. Flow diagram of metabolite extraction from endophytes.


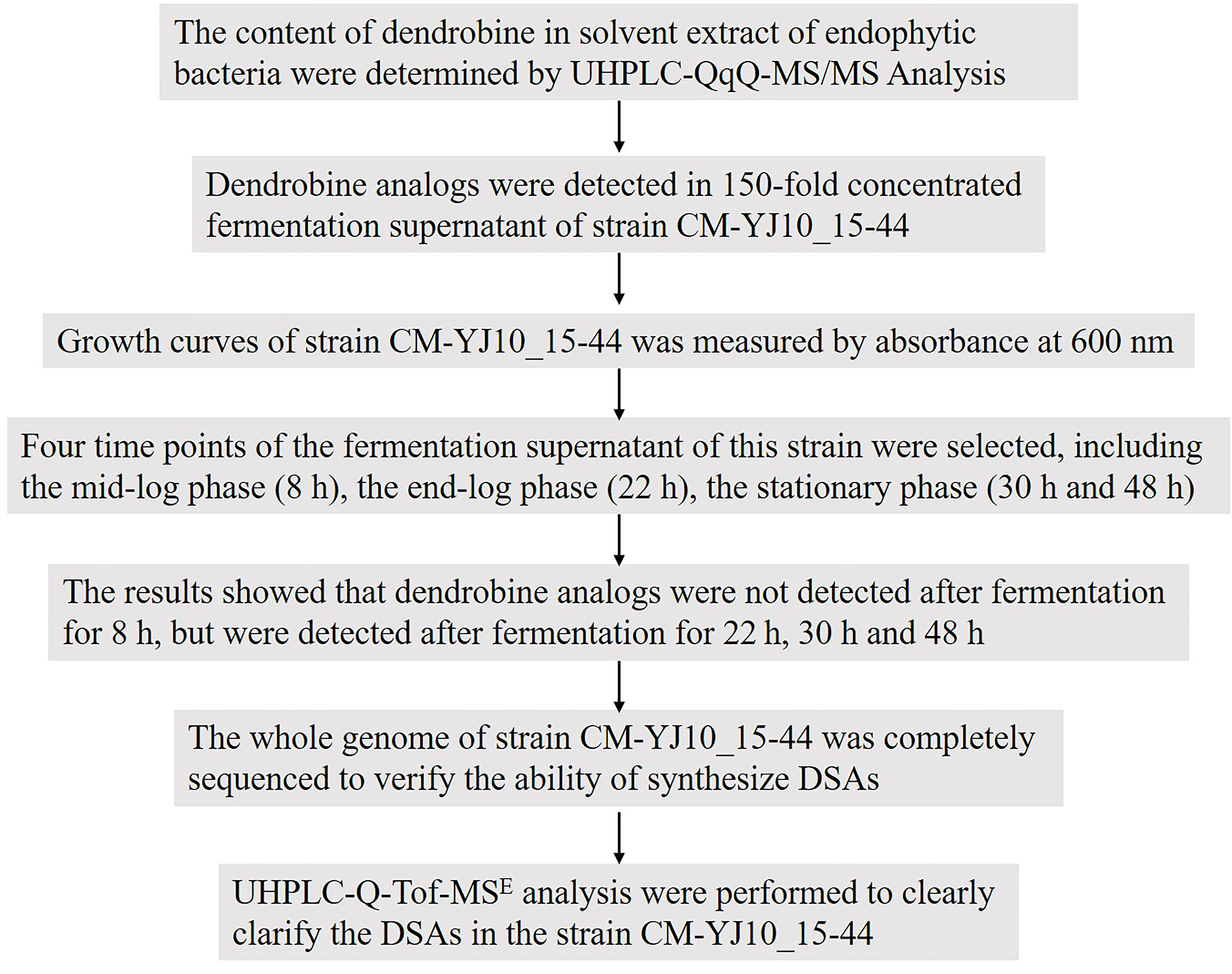


Supplementary Figure 5. Flow diagram of identification of DSAs (6-hydroxydendrobine and nobilonine) from the fermentation supernatant of the strain CM-YJ10_15-44.


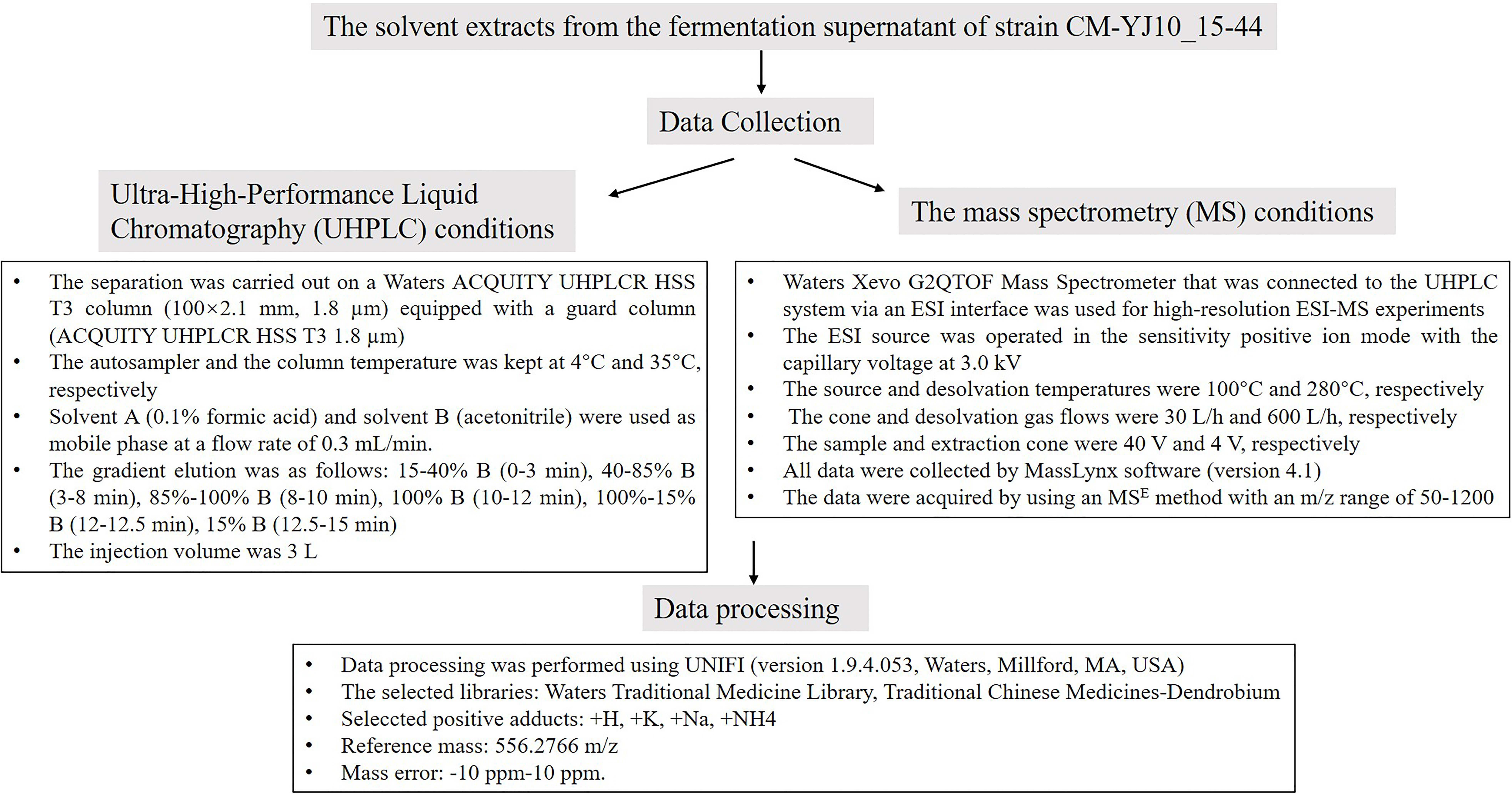


Supplementary Figure 6. Flow diagram of UHPLC-Q-Tof-MS^E^ analysis of DSAs.


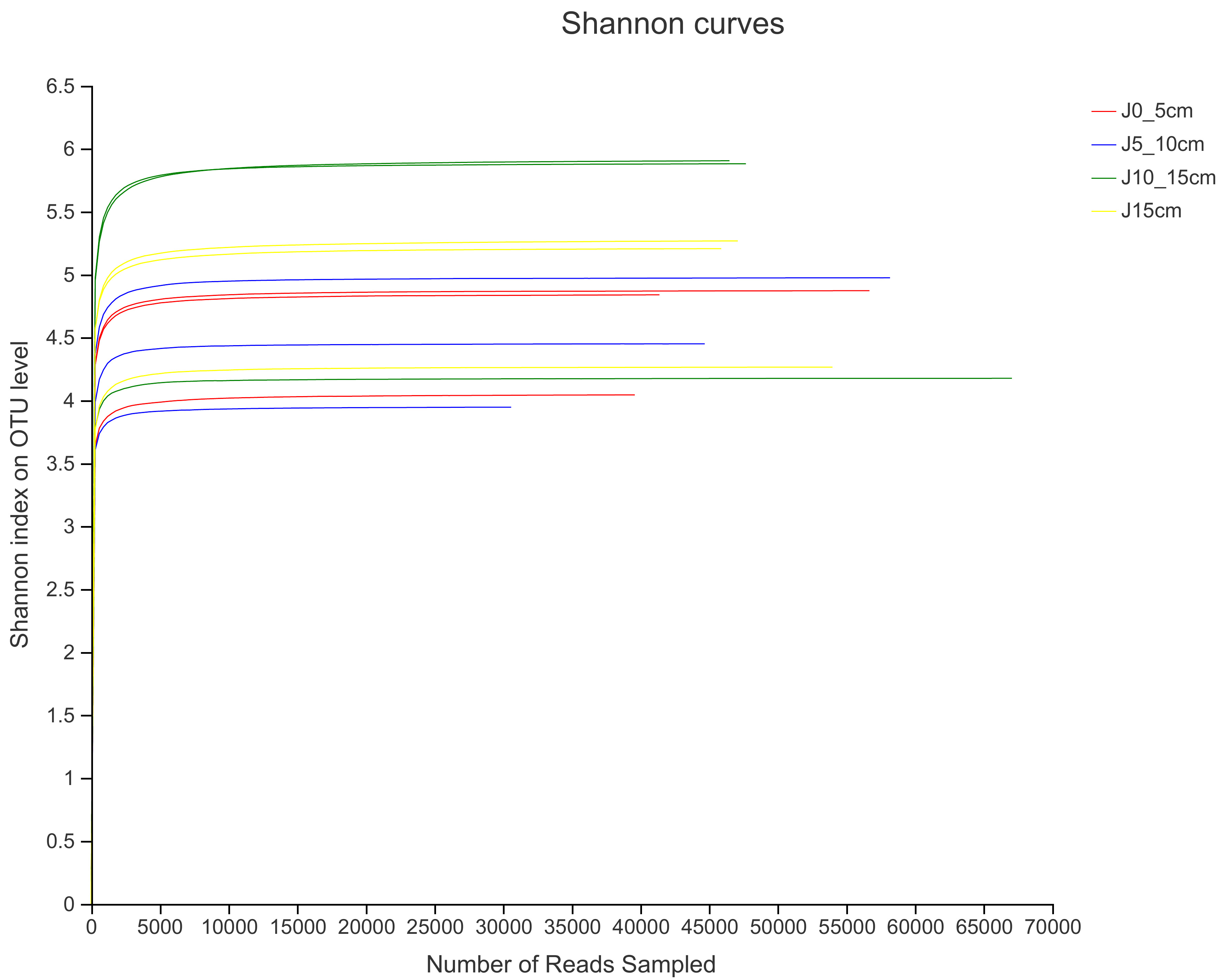


Supplementary Figure 7. Rarefaction curves of the OTU number at 97% similarity for different samples. The rarefaction curves showed that the sequencing work was relatively comprehensive in covering the bacterial diversity, as the curves tended to approach saturation, indicating that the selected sequence data adequately reflected the bacterial abundance of these samples. (Group J0_5cm represents the length of stems was from 0 to 5cm; Group J5_10cm represents the length of stems was from 5 to 10cm; Group J10_15cm represents the length of stems was from 10 to 15cm; Group J15cm represents the length of stems was longer than 15cm.)


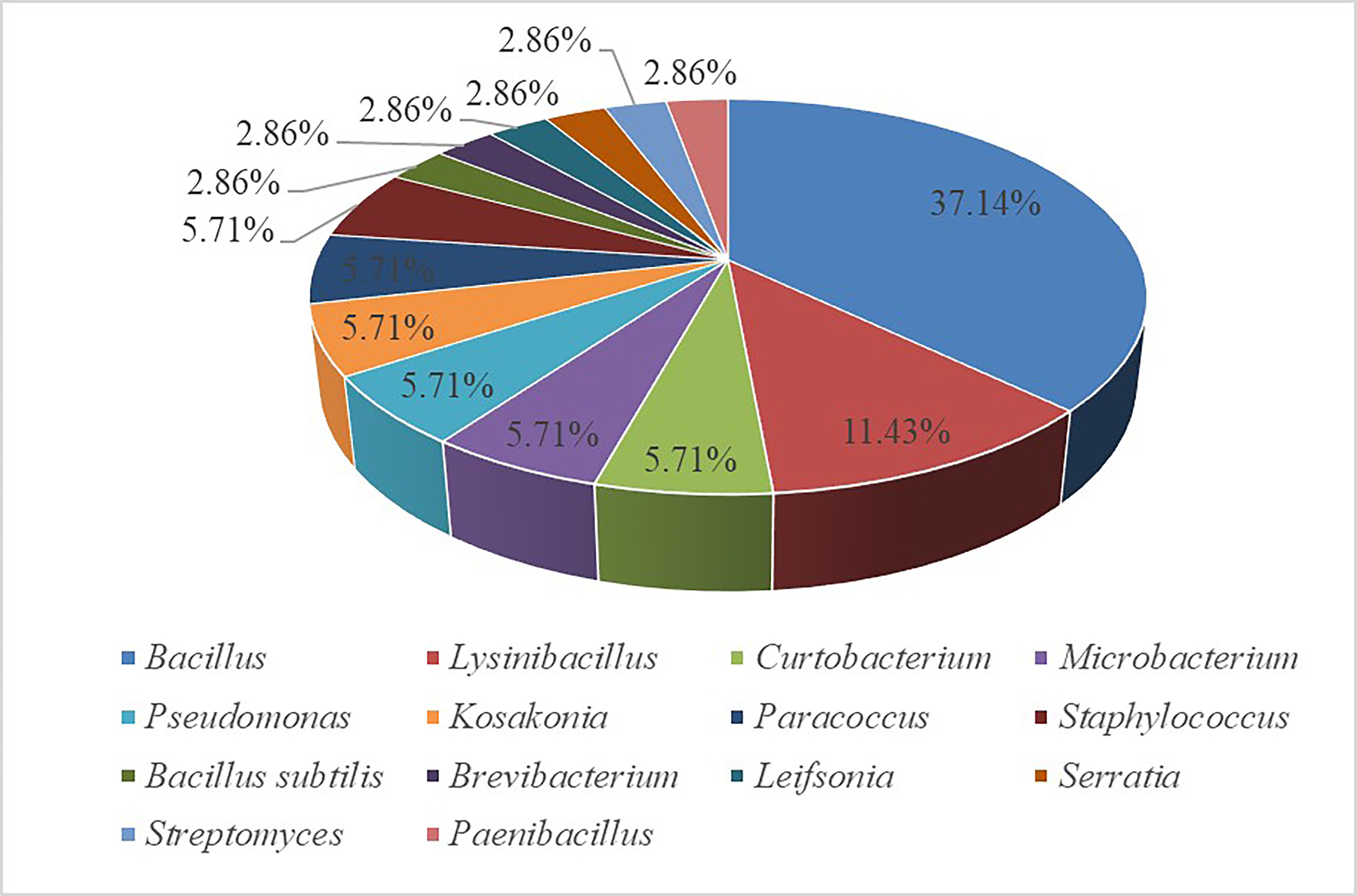


Supplementary Figure 8. Percent of cultivable endophytes on genus level.


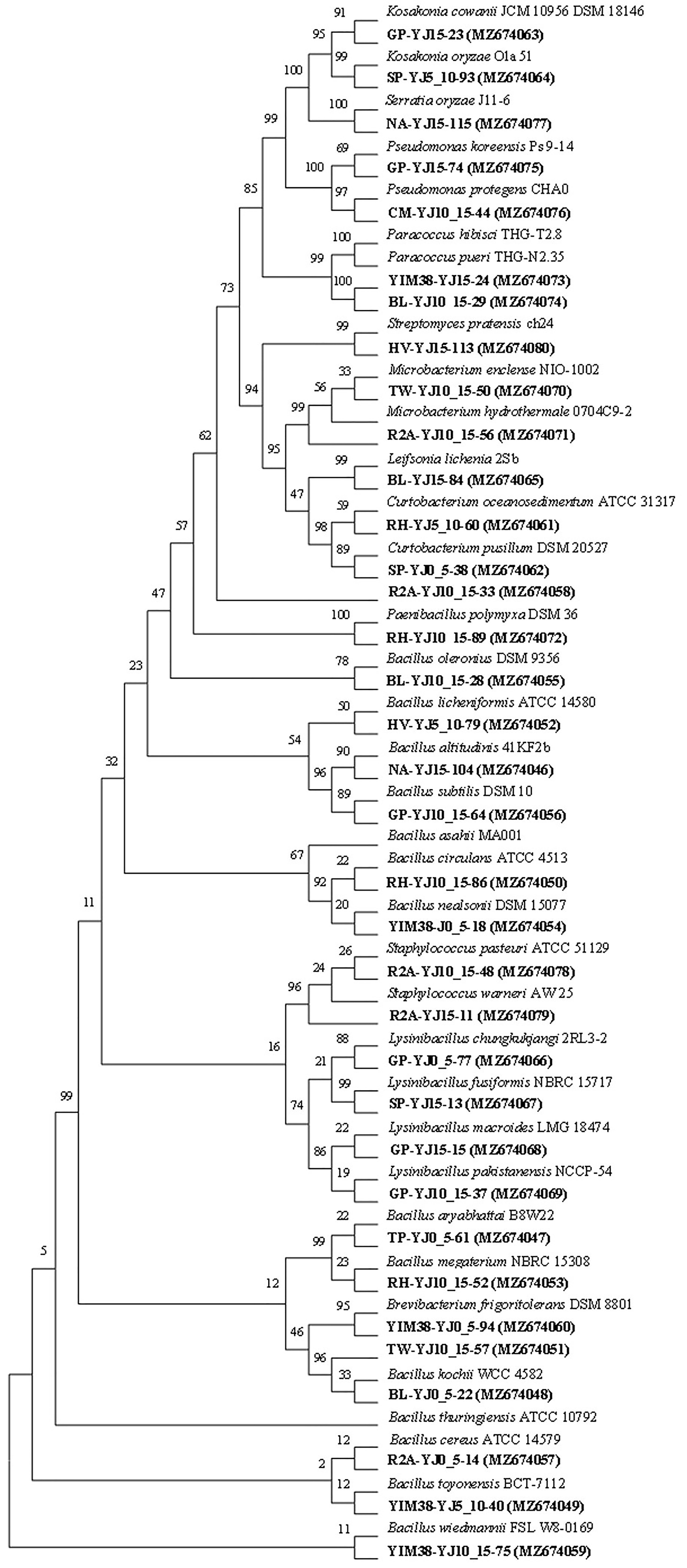


Supplementary Figure 9. The tree of the 35 strains identified in this study. The tree was constructed with the neighbor-joining method with 1000 bootstrap replicates. GenBank accession numbers were given in parentheses. Strain name: Type of medium-YJ stem length-strain number.





Supplementary Figure 10. KEGG functional classification ofprotein sequence in the strain CM-YJ10_15-44 genome. The distributions of the predicted proteins were assigned by the KEGG database. The number of sequences assigned to each sub-category of the Six top KEGG Orthology (KO) categories, namely cellular process (dark blue), metabolism (purple), human diseases (green), genetic information processing (red), organismal systems (orange) and environmental information processing (light blue) were calculated and displayed.


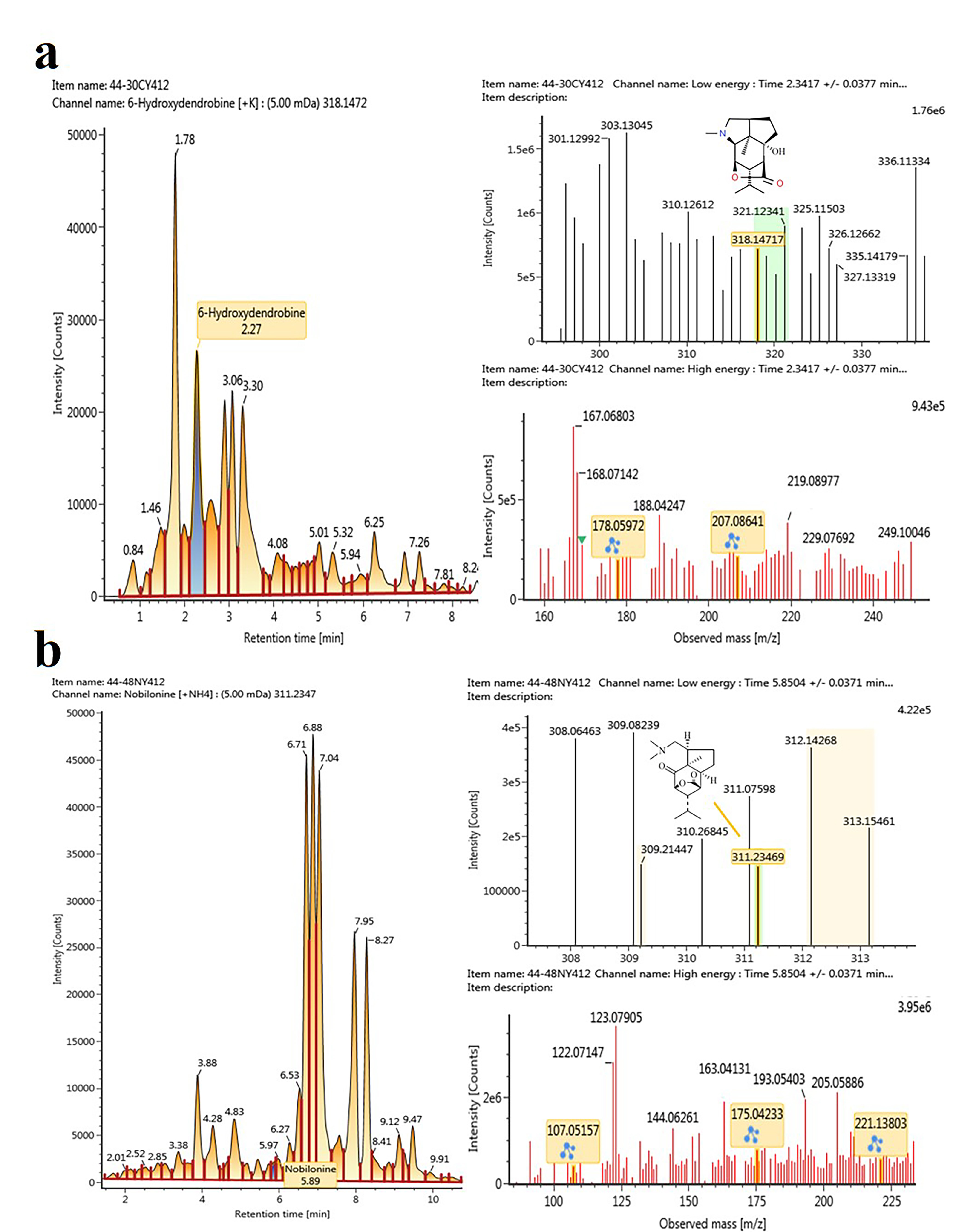


Supplementary Figure 11. (A) Chromatogram, low fragmentation energy mass spectrometry and high fragmentation energy mass spectrometry of Compound 1. (B) Chromatogram, low fragmentation energy mass spectrometry and high fragmentation energy mass spectrometry of Compound 2. (44_30CY412 represents culture supernatant of strain CM-YJ10_15-44 after fermentation for 30 h; 44_48NY412 represents culture supernatant of strain CM-YJ10_15-44 after fermentation for 48 h.)


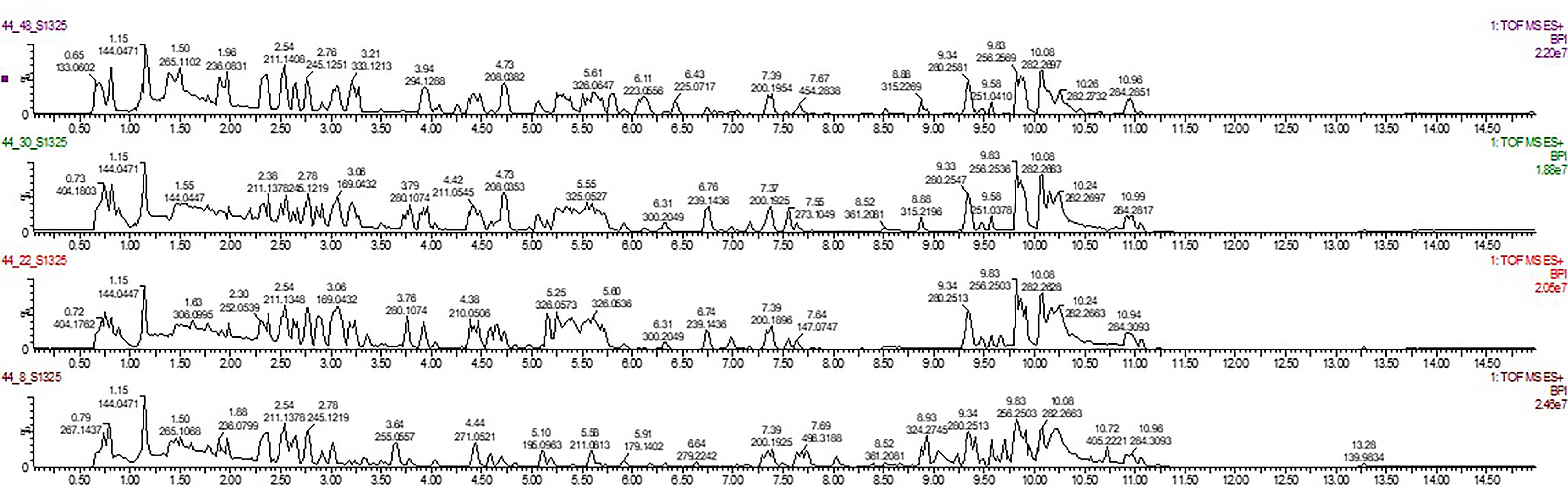


Supplementary Figure 12. The base peak intensity (BPI) chromatograms at positive ion modes of the fermentation supernatant of this strain CM-YJ10_15-44 at different time points. (44_48_S1325 represents culture supernatant of strain CM-YJ10_15-44 after fermentation for 48 h; 44_30_S1325 represents culture supernatant of strain CM-YJ10_15-44 after fermentation for 30 h; 44_22_S1325 represents culture supernatant of strain CM-YJ10_15-44 after fermentation for 22 h; 44_8_S1325 represents culture supernatant of strain CM-YJ10_15-44 after fermentation for 8 h.)

Supplementary Table 1. 11 different media and medium component used in this study.

| **Medium Name** | **Medium Component** |
| --- | --- |
| Humic acid agar (HV) | CaCO_3_ 0.02 g, Humic acid 1.0 g, KCl 1.7 g, FeSO_4_•7H_2_O 0.01 g, MgSO_4_•7H_2_O 0.05 g, Na_2_HPO_4_ 0.5 g, Agar 20.0 g, ultrapure water 1 L, PH:7.2~7.4 |
| YIM38 agar (YIM38) | Glucose 4 g, Yeast extract 4 g, Malt extract 5 g, B-Vitamins 1 mL / L, Trace salt 1 mL / L, Agar 20.0 g, ultrapure water 1 L, PH:7.2~7.4 |
| Tap water yeast glucose starch agar (TW) | Yeast extract 0.25 g, K_2_HPO_4_ 0.5 g, Agar 20.0 g, Tap water 1 L, PH:7.2~7.4 |
| Glucose starch agar (BL) | Glucose 5.0 g, Yeast extract 5.0 g, Soluble starch 5.0 g, Casamino acids hydrolysate 2.0 g, CaCO_3_ 5.0 g, NaCl 5.0 g, Agar 20.0 g, ultrapure water 1 L, PH:7.2~7.4 |
| Sodium propionate agar (SP) | CaCl_2_•2H_2_O 0.02g, Sodium propionate 1.0 g, L-Asparagine 0.2 g, K_2_HPO_4_ 0.6 g, KH_2_PO_4_ 0.9 g, MgSO_4_•7H_2_O 0.1 g, Agar 20.0 g, ultrapure water 1 L, PH:7.2~7.4 |
| Trehalose – proline agar (TP) | CaCl_2_ 2.0 g, (NH_4_)_2_SO_4_ 1.0 g, Trehalose 5.0 g, Proline1.0 g, NaCl 1.0 g, K_2_HPO_4_ 1.0 g, MgSO_4_•7H_2_O 1.0 g, B-Vitamins 1 mL, Agar 20.0 g, ultrapure water 1 L, PH:7.2~7.4 |
| Cellulose agar (CM) | CaCO_3_ 0.02 g, KNO_3_ 0.2 g, Cellulose 10.0 g, Casein 0.3 g, K_2_HPO_4_ 0.2 g, FeSO_4_ 0.01 g, MgSO_4_•7H_2_O 0.05 g, Agar 20.0 g, ultrapure water 1 L, PH:7.2~7.4 |
| M-WA agar (GP) | Glycerol 10.0 g, Yeast extract 0.5 g, KNO_3_ 0.5 g, Proline1.0 g, L-Asparagine 1.0 g, Sodium pyruvate 1.25 g, Betaine 1.25 g, Agar 20.0 g, ultrapure water 1 L, PH:7.2~7.4 |
| 10% Nutrient agar (NA) | Beef extract 0.5 g, Peptone 1 g, Sodium pyruvate 1.25 g, Betaine 1.25 g, NaCl 10 g, Agar 20.0 g, ultrapure water 1 L, PH:7.2~7.4 |
| Raffinose–histidine agar (RH) | Raffinose 1.0 g, CaCO_3_ 0.02 g, Histidine 0.1 g, Na_2_HPO_4_ 0.5 g, KCl 1.7 g, MgSO_4_•7H_2_O 0.05 g, FeSO_4_•7H_2_O 0.1 g, B-Viramins 1 mL, Agar 20.0 g, ultrapure water 1 L, PH:7.2~7.4 |
| R2A agar (R2A) | Glucose 0.5 g, Yeast extract 0.5 g, Peptone 0.5 g, Casein peptone 0.5g, Sodium pyruvate 0.3g, MgSO_4_•7H_2_O 0.024 g, K_2_HPO_4_ 0.3 g, Soluble starch 0.5g, Agar 20.0 g, ultrapure water 1 L, PH:7.2~7.4 |
| The method of adding plant extracts of *Dendrobium* stems to medium as follow: 10 gstems were squeezed in 100 mL of ultrapure water and filtered with four to six layers of gauze. 10mL plant extracts of stems were added to the 1L medium. | |

Supplementary Table 2. Alpha-diversity analyses of endophytes in different *Dendrobium* stems. (Group J0_5cm represents the length of stems was from 0 to 5cm; Group J5_10cm represents the length of stems was from 5 to 10cm; Group J10_15cm represents the length of stems was from 10 to 15cm; Group J15cm represents the length of stems was longer than 15cm.)

| **Group** | **Sample** | **Alpha diversity** | | | | | |
| --- | --- | --- | --- | --- | --- | --- | --- |
|  |  | **Shannon** | **Sobs** | **Simpson** | **Chao** | **Ace** | **Coverage** |
| J0_5cm | J0_5cm1 | 4.4576 | 834 | 0.0279 | 910.5000 | 913.9151 | 99.76% |
|  | J0_5cm2 | 4.9729 | 651 | 0.0182 | 664.6364 | 660.8684 | 99.96% |
|  | J0_5cm3 | 4.4484 | 406 | 0.0362 | 423.2500 | 421.8763 | 99.95% |
| J5_10cm | J5_10cm1 | 4.8375 | 662 | 0.0191 | 708.4063 | 686.4310 | 99.87% |
|  | J5_10cm2 | 4.0428 | 646 | 0.0620 | 725.1507 | 713.6255 | 99.73% |
|  | J5_10cm3 | 4.8705 | 715 | 0.0498 | 751.9643 | 731.8327 | 99.92% |
| J10_15cm | J10_15cm1 | 5.8793 | 1007 | 0.0059 | 1078.0909 | 1036.5880 | 99.86% |
|  | J10_15cm2 | 5.9029 | 1442 | 0.0068 | 1523.7895 | 1506.6474 | 99.68% |
|  | J10_15cm3 | 5.4345 | 1298 | 0.0040 | 1293.6840 | 1181.8395 | 99.82% |
|  | J15cm1 | 5.2047 | 1058 | 0.0113 | 1173.8000 | 1198.2798 | 99.58% |
| J15cm | J15cm2 | 5.2659 | 1232 | 0.0139 | 1413.6142 | 1446.8959 | 99.43% |
|  | J15cm3 | 5.2950 | 1092 | 0.0109 | 979.0976 | 994.2386 | 99.72% |

Supplementary Table 3. Comparison of percentage (%) of the dominant bacterial genera (average abundance >1%) for the 4 groups. (Group J0_5cm represents the length of stems was from 0 to 5cm; Group J5_10cm represents the length of stems was from 5 to 10cm; Group J10_15cm represents the length of stems was from 10 to 15cm; Group J15cm represents the length of stems was longer than 15cm.)

| **Genus** | **J0_5cm** | **J5_10cm** | **J10_15cm** | **J15cm** |
| --- | --- | --- | --- | --- |
| *Acidiphilium* | 4.84 | 2.04 | - | 3.51 |
| *Amnibacterium* | 2.07 | 1.51 | - | 6.10 |
| *Bacillus* | - | - | 1.33 | - |
| *Burkholderia-Paraburkholderia* | 5.27 | 3.99 | - | 2.66 |
| *Bradyrhizobium* | - | - | 2.44 | - |
| *Bryobacter* | - | - | - | 1.85 |
| *Bryocella* | 3.68 | 2.80 | - | 2.11 |
| *Chryseobacterium* | 4.16 | - | - | 7.65 |
| *Frondihabitans* | 2.79 | 2.57 | - | - |
| *Gemmobacter* | - | - | 1.75 | - |
| *Granulicella* | 3.27 | 1.79 | - | - |
| *Jatrophihabitans* | 3.15 | - | - | 2.90 |
| *Kineosporia* | - | - | 2.05 | - |
| *Lactobacillus* | 1.55 | 1.46 | - | - |
| *Massilia* | - | 3.37 | - | - |
| *Methylocella* | 2.70 | 1.53 | - | 2.03 |
| *Methylobacterium* | 6.85 | 4.80 | 1.90 | 6.24 |
| *Mycobacterium* | - | - | 1.73 | - |
| *Pantoea* | 1.51 | - | - | - |
| *Paracoccus* | - | - | 1.16 | - |
| *Pseudonocardia* | - | - | 2.95 | - |
| *Rhodanobacter* | - | 1.23 | - | - |
| *Rhodococcus* | 2.45 | 8.63 | 4.39 | 1.87 |
| *Ralstonia* | - | - | 2.29 | - |
| *Sphingomonas* | 6.22 | 12.39 | 2.66 | 3.90 |
| *Terriglobus* | 2.78 | 1.43 | - | - |
| *Variovorax* | - | - | 1.17 | 1.60 |
| *Norank_f_Acetobacteraceae* | - | - | 1.56 | - |
| *Norank_o_Armatimonadales* | - | - | - | 2.01 |
| *Norank_f_Caulobacteraceae* | 4.93 | 4.31 | - | 5.83 |
| *Norank_f_Muribaculaceae* | 2.93 | 3.68 | - | - |
| *Norank_f_Burkholderiaceae* | - | 1.64 | - | 2.55 |
| *Norank_f_Roseiflexaceae* | - | - | 1.94 | - |
| *Unclassified_f_Acetobacteraceae* | 2.00 | 1.99 | 2.84 | 2.54 |
| *Unclassified_f_Beijerinckiaceae* | 2.23 | - | 1.26 | 2.16 |
| *Unclassified_f_Burkholderiaceae* | - | - | 2.20 | 2.48 |
| *Unclassifiedd_f_Caulobacteraceae* | - | - | - | 1.92 |
| others | 19.32 | 22.41 | 50.35 | 22.41 |

Supplementary Table 4. Details of the 35 strains identified in this study.

| **Strain Number** | **GenBank accession number** | **Closest species in 16S rRNA gene sequences database** | **Similarity**  **(%)** | **Groups** | **Separation medium** |
| --- | --- | --- | --- | --- | --- |
| NA-YJ15-104 | MZ674046 | *Bacillus altitudinis*41KF2b | 99.20 | Group J15cm | NA |
| TP-YJ0_5-61 | MZ674047 | *Bacillus aryabhattai* B8W22 | 99.76 | Group J0_5cm | TP |
| BL-YJ0_5-22 | MZ674048 | *Bacillus asahii*MA001 | 99.48 | Group J0_5cm | BL |
| YIM38-YJ5_10-40 | MZ674049 | *Bacillus cereus*ATCC 14579 | 99.63 | Group J5_10cm | YIM38 |
| RH-YJ10_15-86 | MZ674050 | *Bacillus circulans*ATCC 4513 | 98.62 | Group J10_15cm | RH |
| TW-YJ10_15-57 | MZ674051 | *Bacillus kochii*WCC 4582 | 99.50 | Group J10_15cm | TW |
| HV-YJ5_10-79 | MZ674052 | *Bacillus licheniformis*ATCC 14580 | 98.81 | Group J5_10cm | HV |
| RH-YJ10_15-52 | MZ674053 | *Bacillus megaterium*NBRC 15308 | 98.59 | Group J10_15cm | RH |
| YIM38-J0_5-18 | MZ674054 | *Bacillus nealsonii*DSM 15077 | 98.74 | Group J0_5cm | YIM38 |
| BL-YJ10_15-28 | MZ674055 | *Bacillus oleronius*DSM 9356 | 98.82 | Group J10_15cm | BL |
| GP-YJ10_15-64 | MZ674056 | *Bacillus subtilis* DSM 10 | 99.93 | Group J10_15cm | GP |
| R2A-YJ0_5-14 | MZ674057 | *Bacillus thuringiensis*ATCC 10792 | 99.32 | Group J0_5cm | R2A |
| R2A-YJ10_15-33 | MZ674058 | *Bacillus toyonensis*BCT-7112 | 99.42 | Group J10_15cm | R2A |
| YIM38-YJ10_15-75 | MZ674059 | *Bacillus wiedmannii*FSL W8-0169 | 99.46 | Group J10_15cm | YIM38 |
| YIM38-YJ0_5-94 | MZ674060 | *Brevibacteriumfrigoritolerans*DSM 8801 | 99.01 | Group J0_5cm | YIM38 |
| RH-YJ5_10-60 | MZ674061 | *Curtobacteriumoceanosedimentum*ATCC 31317 | 99.21 | Group J5_10cm | RH |
| SP-YJ0_5-38 | MZ674062 | *Curtobacteriumpusillum* DSM 20527 | 98.32 | Group J0_5cm | SP |
| GP-YJ15-23 | MZ674063 | *Kosakoniacowanii*JCM 10956 | 98.14 | Group J15cm | GP |
| SP-YJ5_10-93 | MZ674064 | *Kosakoniaoryzae*Ola 51 | 98.82 | Group J5_10cm | SP |
| BL-YJ15-84 | MZ674065 | *Leifsonialichenia*2Sb | 98.30 | Group J15cm | BL |
| GP-YJ0_5-77 | MZ674066 | *Lysinibacilluschungkukjangi*2RL3-2 | 98.76 | Group J0_5cm | GP |
| SP-YJ15-13 | MZ674067 | *Lysinibacillus fusiformis*NBRC 15717 | 98.71 | Group J15cm | SP |
| GP-YJ15-15 | MZ674068 | *Lysinibacillusmacrolides* LMG 18474 | 98.91 | Group J15cm | GP |
| GP-YJ10_15-37 | MZ674069 | *Lysinibacilluspakistanensis*NCCP-54 | 100 | Group J10_15cm | GP |
| TW-YJ10_15-50 | MZ674070 | *Microbacteriumhydrothermale*0704C9-2 | 100 | Group J10_15cm | TW |
| R2A-YJ10_15-56 | MZ674071 | *Microbacteriumenclense* NIO-1002 | 99.20 | Group J10_15cm | R2A |
| RH-YJ10_15-89 | MZ674072 | *Paenibacilluspolymyxa* DSM 36 | 99.34 | Group J10_15cm | RH |
| YIM38-YJ15-24 | MZ674073 | *Paracoccushibisci*THG-T2.8 | 98.80 | Group J15cm | YIM38 |
| BL-YJ10_15-29 | MZ674074 | *Paracoccuspueri*THG-N2.35 | 98.98 | Group J10_15cm | BL |
| GP-YJ15-74 | MZ674075 | *Pseudomonas koreensis*Ps 9-14 | 98.80 | Group J15cm | GP |
| CM-YJ10_15-44 | MZ674076 | *Pseudomonas protegens*CHA0 | 99.24 | Group J10_15cm | CM |
| NA-YJ15-115 | MZ674077 | *Serratia oryzae*J11-6 | 99.45 | Group J15cm | NA |
| R2A-YJ10_15-48 | MZ674078 | *Staphylococcus pasteuri*ATCC 51129 | 99.68 | Group J10_15cm | R2A |
| R2A-YJ15-11 | MZ674079 | *Staphylococcus warneri* AW 25 | 99.89 | Group J15cm | R2A |
| HV-YJ15-113 | MZ674080 | *Streptomyces pratensis* ch24 | 98.83 | Group J15cm | HV |

Supplementary Table 5. General features of the genomes of the strain CM-YJ10_15-44.

| **Sample Name** | **Genome size (bp)** | **GC content (%)** | **CDS No.** | **tRNA No.** | **rRNA No.** | **Gene average length (bp)** | **Gene density** |
| --- | --- | --- | --- | --- | --- | --- | --- |
| CM-YJ10_15-44 | 6953246 | 63.36 | 6387 | 71 | 16 | 959.16 | 0.92 |

Supplementary Table 6. Secondary metabolites biosynthetic gene clusters of strain CM-YJ10_15-44 with the similarity more than 20%.

| **Cluster ID** | **Type** | **Start** | **End** | **Similar Cluster** | **Similarity**  **(%)** | **Gene No.** |
| --- | --- | --- | --- | --- | --- | --- |
| Cluster4 | T1pks | 3196682 | 3249224 | Pyoluteorin_biosynthetic_gene_cluster,polyketide | 100 | 44 |
| Cluster6 | other | 4144297 | 4185382 | Pyrrolnitrin_biosynthetic_gene_cluster,other | 100 | 35 |
| Cluster10 | T3pks | 6625367 | 6666389 | 2,4-Diacetylphloroglucinol_biosynthetic_gene_cluster, polyketide | 100 | 41 |
| Cluster3 | Nrps | 2459136 | 2533673 | Orfamide_biosynthetic_gene_cluster,nrps | 94 | 49 |
| Cluster1 | arylpolyene | 509656 | 553271 | APE_Vf_biosynthetic_gene_cluster,other | 40 | 44 |
| Cluster7 | Nrps | 4735780 | 4799967 | Pyoverdine_biosynthetic_gene_cluster,nrps | 29 | 38 |
| Cluster5 | Nrps | 4012035 | 4066081 | Coelibactin_biosynthetic_gene_cluster,nrps | 27 | 43 |
| Cluster8 | Nrps | 4866603 | 4919629 | Pyoverdine_biosynthetic_gene_cluster,nrps | 22 | 41 |
